# Disruption of Proteostasis by Natural Products and Synthetic Compounds That Induce Pervasive Unfolding of Proteins: Therapeutic Implications

**DOI:** 10.3390/ph16040616

**Published:** 2023-04-19

**Authors:** Nuria Vilaboa, Juan Antonio Lopez, Marco de Mesa, Clara Escudero-Duch, Natalie Winfield, Melanie Bayford, Richard Voellmy

**Affiliations:** 1 Hospital Universitario La Paz-IdiPAZ, 28046 Madrid, Spain; 2 CIBER de Bioingenieria, Biomateriales y Nanomedicina, CIBER-BBN, 28046 Madrid, Spain; 3 Centro Nacional de Investigaciones Cardiovasculares, CNIC, 28029 Madrid, Spain; 4 CIBER de Enfermedades Cardiovasculares, CIBERCV, 28029 Madrid, Spain; 5 Domainex Ltd., Chesterford Research Park, Little Chesterford, Essex, Saffron Walden CB10 1XL, UK; 6 HSF Pharmaceuticals SA, 1814 La Tour-de-Peilz, Switzerland

**Keywords:** proteostasis inhibition, proteostasis disruption, protein unfolding, protein aggregation, IHSF, celastrol, withaferin A, multiple myeloma 1

## Abstract

Exposure of many cancer cells, including multiple myeloma cells, to cytotoxic concentrations of natural products celastrol and withaferin A or synthetic compounds of the IHSF series resulted in denaturation of a luciferase reporter protein. Proteomic analysis of detergent-insoluble extract fractions from HeLa-derived cells revealed that withaferin A, IHSF058 and IHSF115 caused denaturation of 915, 722 and 991 of 5132 detected cellular proteins, respectively, of which 440 were targeted by all three compounds. Western blots showed that important fractions of these proteins, in some cases approaching half of total protein amounts, unfolded. Relatively indiscriminate covalent modification of target proteins was observed; 1178 different proteins were modified by IHSF058. Further illustrating the depth of the induced proteostasis crisis, only 13% of these proteins detectably aggregated, and 79% of the proteins that aggregated were not targets of covalent modification. Numerous proteostasis network components were modified and/or found in aggregates. Proteostasis disruption caused by the study compounds may be more profound than that mediated by proteasome inhibitors. The compounds act by a different mechanism that may be less susceptible to resistance development. Multiple myeloma cells were particularly sensitive to the compounds. Development of an additional proteostasis-disrupting therapy of multiple myeloma is suggested.

## 1. Introduction

The metastable proteome is maintained by the protein homeostasis or proteostasis network (“PN”). The PN is a multi-compartment system that comprises the translation machinery, chaperones and co-chaperones; the ubiquitin-proteasome system and the autophagy system [1,2,3]. Much research has been concerned with the maintenance or enhancement of proteostasis to counteract age-related decline and/or to prevent or treat certain diseases that involve protein aggregation. The opposite concept that disruption of proteostasis could be an effective anti-cancer strategy also has been investigated actively for many years.

To date, the only clinically proven treatment strategy based on disruption of proteostasis is inhibition of proteasome activity. Proteasome inhibitors are deployed in the treatment of multiple myeloma (MM) and mantle cell lymphoma but not solid tumors [4,5]. It has been argued that MM cells may be particularly dependent on proteasome function, owing to their excessive synthesis of immunoglobulins, which is inherently accompanied by increased production of unfolded proteins and defective ribosomal products [6]. Another approach, inhibition of major chaperone HSP90, has not produced potent anti-tumor responses in multiple clinical trials [7,8]. The 13 inhibitors investigated all induced a heat shock response that apparently interfered with their clinical activity [9,10,11]. Other avenues that have been proposed include inhibition of HSP70, another major chaperone and inhibition of heat shock factor 1 (HSF1), the master switch of the heat shock response, on which many tumor cells appear to have a heightened dependence [12,13,14,15,16,17].

We describe herein another mechanism of proteostasis inhibition that may be exploited for the therapy of certain human cancers, in particular MM. We found that certain compounds that possess an α,β-unsaturated carbonyl group (a potential Michael acceptor that may interact with protein thiols) but do not share any other discernible structural feature induce massive, generalized protein unfolding at concentrations at which they are cytotoxic, apparently brought about by relatively indiscriminate covalent modification of cellular proteins. The compounds studied were synthetic compounds referred to as the IHSF series as well as the natural products celastrol (CEL) and withaferin A (WA). IHSF series compounds have been explored previously as inhibitors of HSF1 [18]. One of these compounds, IHSF115, was found to have activity in a PC-3 prostate cancer xenograft model [19]. CEL, produced by “The Thunder of God” vine Tripterygium wilfordii, was investigated extensively for its activity against obesity, inflammatory, auto-immune and neurodegenerative diseases as well as various cancers [20,21,22,23,24,25,26,27,28,29,30,31,32,33,34,35]. WA, found in the Ashwagandha plant Withania somnifera, was reported to have cytoprotective, anti-inflammatory and anti-cancer effects [36,37,38,39,40,41,42,43,44,45,46,47]. Diverse specific targets for CEL and WA were proposed [20,21,22,23,24,25,29,30,31,32,33,34,35,38,39,42,43,45,46,47,48,49,50,51,52,53].

Our study documents the proteostasis-disrupting activity of CEL, WA and compounds of the IHSF series and provides a rational underpinning for exploring these compounds as therapeutic agents against multiple myeloma.

## 2. Results

### 2.1. Inactivation of RLUC by IHSF Series Compounds, CEL and WA

**Figure 1:**
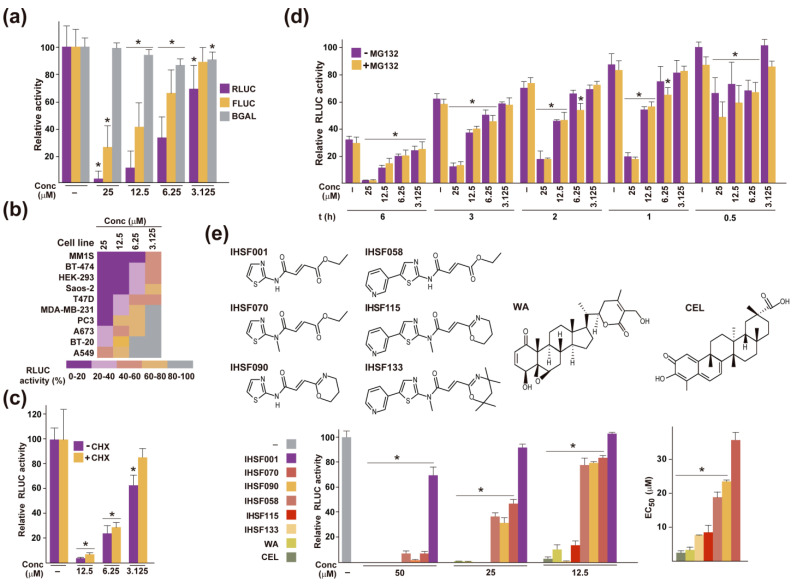
Enhanced inactivation of reporter proteins mediated by IHSF, CEL and WA. (**a**) Parallel cultures of HeLa cells were transfected with reporter constructs pRL-CMV (RLUC), pCMV-Luc (FLUC) or pCMV-βGal (BGAL) using a standard lipofectamine procedure. One day later, the cultures were exposed to the concentrations of IHSF115 indicated in the panel or vehicle (-) for 6 h. Reporter activities were determined as described in Section 4. *: *p* < 0.05 (compared to cells exposed to vehicle). (**b**) Heatmap depicting RLUC activities in the indicated cell lines after 6 h exposures to different concentrations of IHSF115. Cells were transfected one day prior with pRL-CMV, as in (**a**), except that MM1S cells were transduced with adenoviral construct Ad-CMV-RLUC at an MOI of 100. RLUC activities were determined as in (**a**). (**c**) Parallel cultures of HeLa cells previously transfected with pRL-CMV were exposed for 6 h to the indicated concentrations of IHSF115 or vehicle (-) in the presence or absence of 50 µg/mL cycloheximide (CHX). Transfections and reporter activity determinations were performed as in (**a**). *: *p* < 0.05 (compared to cells exposed to vehicle). (**d**) Parallel cultures of HeLa cells previously transfected with pRL-CMV were exposed to the indicated concentrations of IHSF115 or vehicle (-) for the indicated times in the presence or absence of MG-132 (10 µM). Transfections and reporter activity determinations were as in (**a**). *: *p* < 0.05 (compared to cells exposed to vehicle, in the presence or absence of MG-132, at each time point). (**e**) Enhanced inactivation of RLUC caused by different IHSF series compounds, CEL and WA. The structures of the compounds evaluated are depicted at the top. RLUC activities (determined as in (**a**)) in M1 cells after 6 h of exposure to the indicated concentrations of compounds or to the common vehicle are shown in the left graph at the bottom. *: *p* < 0.05 (compared to cells exposed to vehicle). IC50 values (50% reduction in RLUC activity relative to the control after a 6 h exposure) were 67.8 +/− 19.5 µM for IHSF001, 20.4 +/− 7.0 µM for IHSF058, 20.4 +/− 7.3 µM for IHSF070, 16.6 +/− 2.5 µM for IHSF090, 10.0 +/− 0.9 µM for WA, 9.0 +/− 3.8 µM for IHSF115, 4.5 +/− 2.0 µM for CEL and 2.4 +/− 0.5 µM for IHSF133. EC50 values (50% viability after a 96 h of exposure to compound relative to a control exposure to vehicle) estimated by alamar blue assay are shown in the right graph at the bottom. EC50 of IHSF001 could not be calculated as the viability of cells treated at 50 µM was only reduced by 3.8%. *: *p* < 0.05 (compared to cells exposed to IHSF070).

When HeLa cells transfected with a Renilla luciferase (RLUC) expression construct were exposed for 6 h to increasing concentrations of IHSF115, a concentration-dependent decline in RLUC activity was observed (Figure 1a). Inactivation, albeit less pronounced, was also found for firefly luciferase, but not for bacterial β-galactosidase. IHSF115-mediated RLUC inactivation occurred in many different cancer cell lines, most dramatically in the MM cell line MM1S transduced with Ad-CMV-RLUC (Figure 1b). The phenomenon was also observed in the presence of cycloheximide (CHX), demonstrating that it was not dependent on protein synthesis (Figure 1c). Although CHX showed little effect in the experiment, it was included in subsequent exposure experiments to minimize potential confounding effects of protein synthesis (unless indicated otherwise). A time course experiment revealed that the full dose-dependent effect of IHSF115 manifested itself after one hour of exposure (Figure 1d). Thus, the inactivating effect of the IHSF was rapid as well as time-limited. Note that some inactivation of RLUC occurred in the absence of the compound, but the compound greatly enhanced this effect. To determine whether the enhanced inactivation of RLUC could be explained by increased proteasome-mediated degradation, the proteasome inhibitor MG-132 was added to parallel cultures. No significant effect caused by MG-132 was observed.

Accelerated RLUC inactivation was also caused by other members of the IHSF series, CEL and WA (Figure 1e). IC50 values (50% reduction in RLUC activity relative to the vehicle (DMSO) control after a 6 h of exposure) differed considerably between the compounds (Figure 1e, bottom left). Note that these and many of the subsequent experiments were carried out using HeLa-derived cell line M1 that contained a stably transfected gene for RLUC. Cytotoxicity of the compounds generally paralleled their RLUC-inactivating activity (Figure 1e, bottom right). EC50 values (50% viability after a 96 h exposure to compound) are shown.

### 2.2. IHSF Series Compounds, CEL and WA Cause RLUC and Cellular Proteins to Unfold

**Figure 2:**
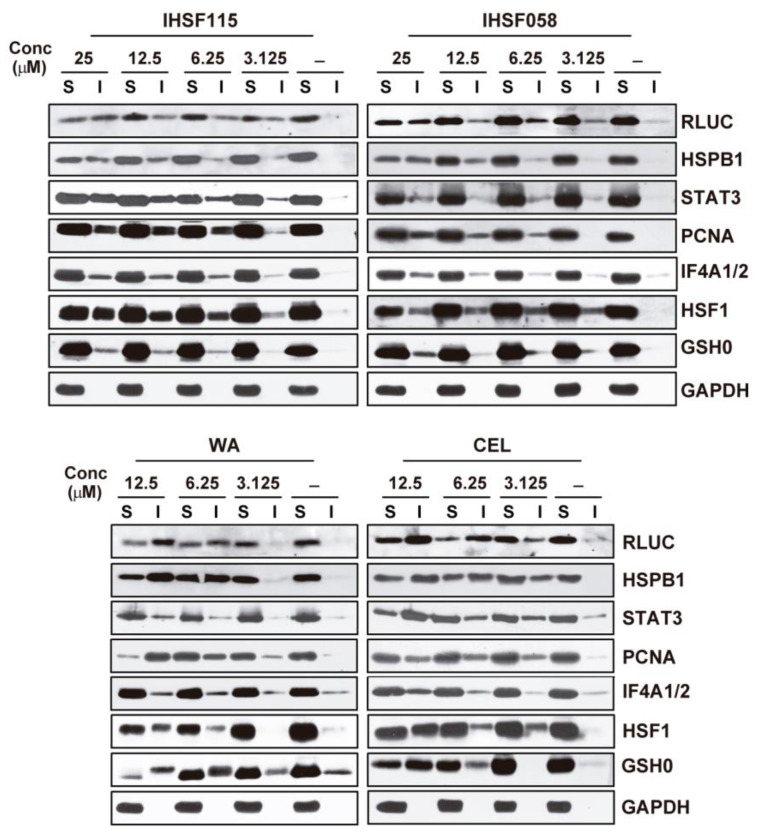
Denaturation of selected proteins in M1 cells exposed to increasing concentrations of IHSF115, IHSF058, CEL or WA. Parallel cultures of M1 cells exposed to compounds or vehicle (-) for 6 h were harvested, extracts were prepared, and the extracts were separated into detergent-soluble (S) and detergent-insoluble (I) fractions as described in the Section 4. Extract fractions (25 µg of protein as determined by a Bradford-based assay) were analyzed via WB using the antibodies specified in the Section 4.

M1 cells were exposed to different concentrations of IHSF058, IHSF115, CEL and WA. Extracts were separated into detergent-soluble and detergent-insoluble fractions that were analyzed via RLUC WB. All compounds caused pronounced changes in the distribution of RLUC between detergent-soluble and detergent-insoluble fractions (Figure 2). While normally present mostly in the soluble fractions, RLUC was found to be dramatically enriched in the detergent-insoluble fractions of cells exposed to elevated concentrations of the compounds. About half of the total protein amount (or more) was present in the detergent-insoluble fractions. Less dramatic changes in distribution were observed at lower compound concentrations. Thus, all compounds promoted the unfolding of RLUC.

The redistribution of several cellular proteins was also analyzed via WB (Figure 2). Large proportions of some of these proteins were found in the detergent-insoluble fractions of cells exposed to elevated concentrations of the compounds. Accumulation of the proteins in the detergent-insoluble fractions was already apparent at compound concentrations of 3.125 or 6.25 µM. Hence, the compounds also caused substantial unfolding of cellular proteins. GAPDH was included in the experiments as an example of a protein that does not unfold.

**Figure 3:**
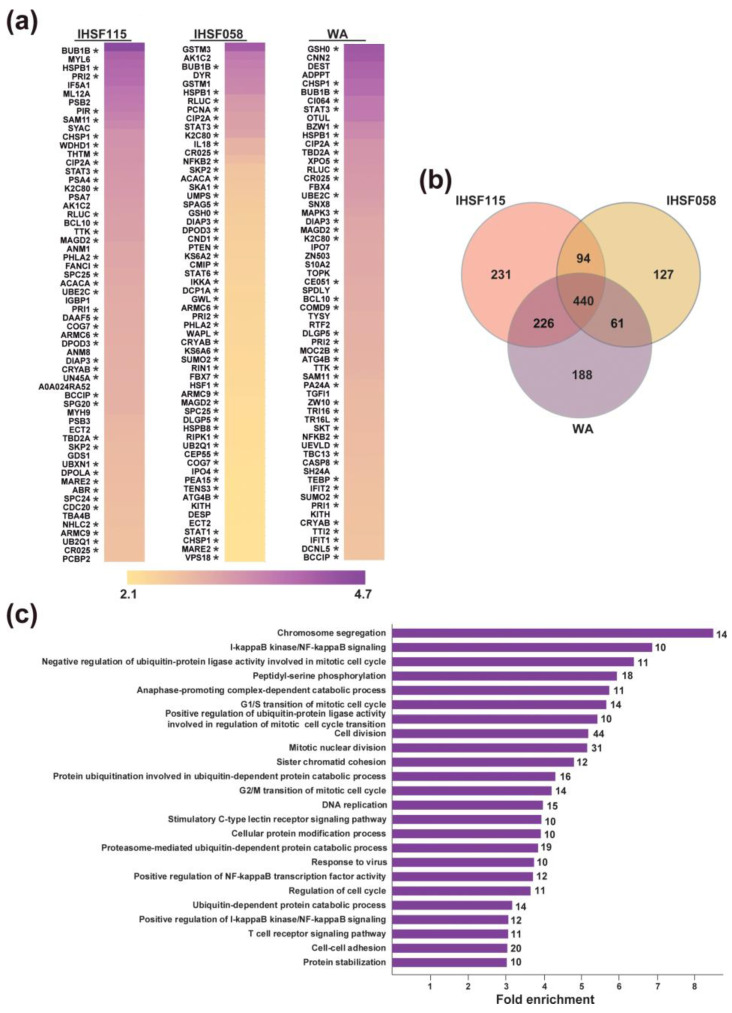
Proteomic analysis of compound-induced protein unfolding. (**a**) Parallel cultures of M1 cells were exposed to 25 µM IHSF115, 25 µM IHSF058, 12.5 µM WA or vehicle for 6 h. Detergent-insoluble fractions of extracts were analyzed using TMT mass spectrometry. The heatmaps provide, for each of the compounds, the 60 proteins whose relative amounts increased the most in the detergent-insoluble extract fractions. Asterisks identify proteins commonly aggregating in cells exposed to IHSF115, IHSF058 and WA. (**b**) Venn diagram showing the overlap between proteins that were induced to unfold by each of the compounds. (**c**) Selected results of a GO analysis of the 440 commonly denatured proteins. Complete results are found in Table S5.

M1 cells were exposed to 25 µM IHSF115, 25 µM IHSF058, 12.5 µM WA or vehicle for 6 h. Detergent-insoluble extract fractions were analyzed using TMT mass spectrometry (Tables S1–S3). For each experimental condition, proteins from three or more independent cultures were analyzed. Proteins were identified as having been induced to denature if their relative abundances increased at least 1.5-fold in the detergent-insoluble extract fractions of compound-treated cells. Commonly detected in these experiments were 5132 cellular proteins. In cells exposed to IHSF115, 991 proteins accumulated significantly in the detergent-insoluble fractions. WA induced 915 proteins to denature and IHSF058 722 proteins. The heatmaps in Figure 3a report on the 60 proteins (including RLUC) that aggregated most dramatically in cells exposed to the different compounds. The Venn diagram in Figure 3b shows that 440 cellular proteins were induced to unfold by all three compounds (“commonly unfolding proteins”, listed in Table S4). Hence, it appears that the target selectivity of the compounds is limited but not entirely absent. This is further illustrated by a comparison of rank orders (Zq values) of accumulating proteins in cells treated with the different compounds (Tables S6–S8). A GO analysis of the 440 commonly denatured proteins revealed substantial enrichment in multiple process categories (Table S5). Many of the well-populated categories that were enriched more than 5-fold related to cell cycling and cell division (Figure 3c). Several categories comprising components of the PN were also substantially enriched.

### 2.3. Proteomic Analyses of Induced Protein Unfolding

**Figure 4:**
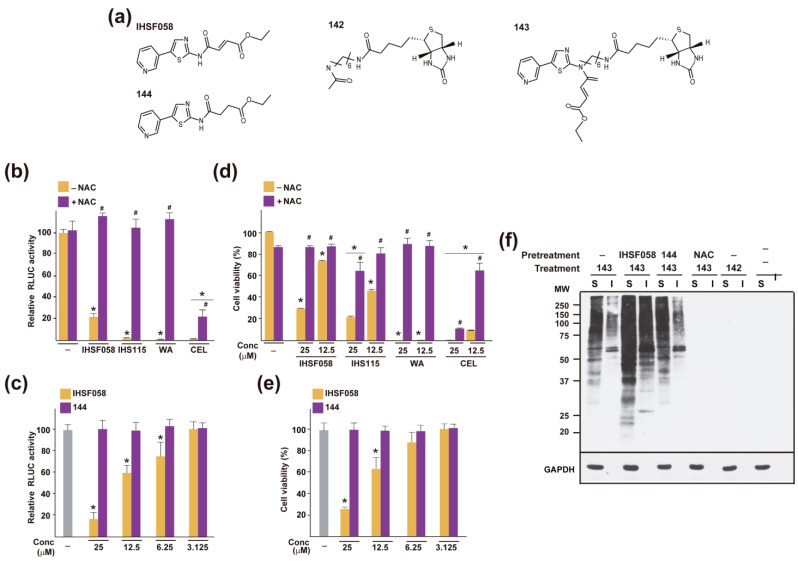
Thiol reactivity of IHSF, CEL and WA, and covalent modification of proteins by biotinylated IHSF058. (**a**) Structures of IHSF058 and compounds 142–144. (**b**) Parallel cultures of M1 cells were exposed for 6 h to vehicle (-) or to 25 µM IHSF058, IHSF115, WA or CEL in the presence or absence of 15 mM NAC. RLUC activities were determined as described in the Section 4. *: *p* < 0.05 (compared to cells exposed to vehicle). #: *p* < 0.05 (compared to cells treated with compound in the absence of NAC). (**c**) Parallel cultures of M1 cells were exposed for 6 h to vehicle (-) or to increasing concentrations of IHSF058 or compound 144. RLUC activities were determined as described in the Section 4. *: *p* < 0.05 (compared to cells exposed to vehicle). (**d**) Parallel cultures of Hela cells were exposed for 96 h to vehicle (-) or the indicated concentrations of IHSF058, IHSF115, WA or CEL in the presence or absence of 5 mM NAC. Viability was estimated via alamar blue assay. *: *p* < 0.05 (compared to cells exposed to vehicle, in the presence or absence of NAC). #: *p* < 0.05 (compared to cells treated with the same dose of compound in the absence of NAC). (**e**) Parallel cultures of Hela cells were exposed for 96 h to vehicle (-) or the indicated concentrations of IHSF058 or compound 144. Viability was estimated as in (d). *: *p* < 0.05 (compared to cells exposed to vehicle). (**f**) Parallel cultures of M1 cells were pre-exposed for 3 h to vehicle (-), 25 µM IHSF058, 25 µM compound 144 or 15 mM NAC. The cultures were thereafter further exposed for 6 h to DMSO, 25 µM compound 143 or 25 µM compound 142. Extracts were prepared and separated into detergent-soluble (S) and detergent-insoluble (I) fractions as described in the Section 4. Aliquots of extract fractions (3 µg protein) were separated on a 12% SDS-PAGE gel. Modified proteins were visualized on blots of the SDS-PAGE gel with streptavidin-HRP. A separate anti-GAPDH WB served as a control for the procedure.

CEL and WA were known to be capable of covalently modifying certain proteins, typically at cysteine residues [33,34,35,38,42,48,52,53,54,55]. As was shown in a model experiment, the thiol reactivity of WA was abolished when the relevant α,β-unsaturated carbonyl group of the compound was reduced [45]. To obtain analogous evidence for IHSF, we inquired whether N-acetylcysteine (NAC) could blunt the activities of IHSF058 and IHSF115 in the RLUC-inactivation assay. Co-exposure of M1 cells to 15 mM NAC completely abolished RLUC inactivation by 25 µM IHSF058 or IHSF115 (Figure 4b). Similar results were obtained for CEL and WA (though, for CEL, the effect of NAC was only partial, perhaps owing to the high activity of the compound). Next, we synthesized compound 144, which is identical to IHSF058 but lacks its α,β-unsaturated carbonyl group (Figure 4a). Compound 144 was unable to enhance RLUC inactivation in M1 cells (Figure 4c). Thus, the α,β-unsaturated carbonyl group present in the IHSF is responsible, directly or indirectly, for the accelerated inactivation of RLUC. NAC (5 mM) essentially abolished the effects of IHSF058, IHSF115, CEL and WA on cell viability (Figure 4d), and compound 144 showed no detectable cytotoxicity (Figure 4e). Mining our mass spectrometric data to identify covalently modified proteins was not successful. Instead, we synthesized compound 143, which is identical to IHSF058, except for an aliphatic side chain terminating in a biotin group (Figure 4a). Compound 142 consisting of the latter substituent served to detect nonspecific interactions. M1 cells were incubated with compound 143, compound 142, or vehicle. Detergent-soluble and detergent-insoluble extract fractions were analyzed with WB. Covalently modified (biotinylated) proteins were visualized using streptavidin-HRP (Figure 4f). Both detergent-soluble and detergent-insoluble fractions of cells incubated with compound 143 but not with compound 142 or vehicle contained multitudes of modified proteins. Pre-incubation with IHSF058 enhanced adduct formation by compound 143, *NAC* prevented it and compound 144 had no effect. These experiments revealed that compound 143, a biotinylated version of IHSF058, extensively modified cellular proteins in situ.

To identify covalently modified proteins, triplicate cultures of M1 cells were incubated with compound 143 or control compound 142, and biotinylated proteins in (unfractionated) extracts were captured using streptavidin beads. Captured proteins were analyzed via mass spectrometry. The experiment detected 1178 proteins that were highly enriched in samples from compound 143-exposed cells (Table S9). Only 150 of the 722 proteins detectably aggregating in IHSF058-exposed cells were found to be modified (Table S10). A GO analysis of the latter 150 proteins is found in Table S11.

### 2.4. Other Shared Properties of CEL, WA and IHSF Series Compounds

**Figure 5:**
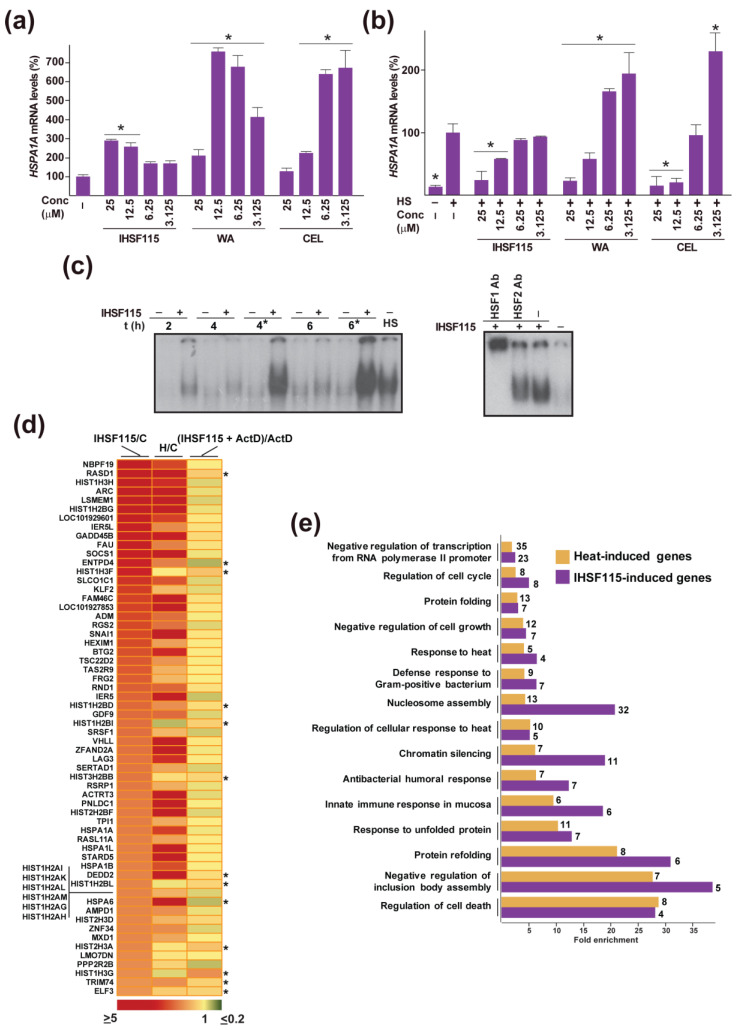
Responses of cells exposed to IHSF, CEL and WA. (**a**) Induction of HSPA1A transcription by CEL, WA and IHSF115. Parallel cultures of HeLa cells were exposed for 2 h to different concentrations of the compounds or vehicle (-), harvested and total RNA was prepared. Relative levels of HSPA1A transcripts were determined through RT-qPCR, all as described in the Section 4. *: *p* < 0.05 (compared to cells exposed to vehicle). (**b**) Inhibition of HSF1 activity. Experiment as in (**a**), except that compound-exposed and control cultures were subjected to a heat treatment (HS) at 43.5 °C for 30 min and then further incubated at 37 °C for 1 h. *: *p* < 0.05 (compared to heat-treated cells exposed to vehicle). (**c**) Enhancement of HSF1 DNA-binding activity by IHSF115. Cultures of HeLa cells were exposed to vehicle (-) or to 25 µM IHSF115 for 2, 4 or 6 h. Additional cultures were similarly exposed for 4 or 6 h, but the medium was replaced with fresh medium containing 25 µM IHSF115 (or vehicle) at 2 h or at 2 h and 4 h, respectively (asterisks). For comparison purposes, control cultures were subjected to a heat treatment (HS) at 43.5 °C for 30 min. EMSA using a radiolabeled HSE probe was carried out as described in the Section 4. Samples from cells exposed to 25 µM IHSF115 for 6 h (medium refreshed after 2 and 4 h) were used for HSF1 supershift. HSF1 or HSF2 antibodies were added to binding reactions prior to electrophoresis. (**d**) Transcriptomic response to IHSF115. RNA prepared from HeLa cells that were exposed for 6 h to 25 µM IHSF115 (medium refreshed after 2 and 4 h) or vehicle (C) was analyzed on Affymetrix GeneChips as described in the Section 4. The ratios of experimental and control signals for the 60 genes whose expression was enhanced the most by compound exposure are shown in the left column of the heatmap. The middle column relates to ratios of experimental (H) and control (C) signals for heat activation of the same genes imported from Ref. [18]. The right column shows ratios comparing transcript levels in cells treated for 1 h with 25 µM IHSF115 and 10 µg/mL actinomycin D (IHSF115 + ActD) versus cells treated with 10 µg/mL actinomycin D alone (ActD). Asterisks mark significant changes (*p* < 0.05) between (IHSF115 + ActD) and ActD values. (**e**) Selected results of a GO analysis of IHSF115- and heat-induced transcripts. Complete results are found in Table S14.

CEL and WA were known to induce HSP expression [45,54]. To confirm and extend this observation to IHSF-series compounds, HeLa cell cultures were exposed for 2 h to CEL, WA or IHSF115. Modestly elevated HSPA1A transcript levels were observed for all three compounds, with CEL and WA being more effective than IHSF115 (Figure 5a). That transcript levels in CEL- or WA-treated cells plateaued and then decreased with increasing compound concentrations suggested that HSF1 transcriptional activity was progressively inhibited. This suggestion was strengthened by the finding that these compounds were capable of strongly inhibiting stress-induced HSPA1A expression at elevated concentrations (Figure 5b). Enhancing effects were seen at low concentrations. Inhibition by IHSF115 increased monotonously with compound concentration, perhaps owing to the fact that the compound has some affinity for HSF1 [18].

That CEL, WA and IHSF115 stimulated HSPA1A expression suggested that the compounds provoked a transcriptional response resembling a heat shock response. We explored this hypothesis for IHSF115. First, we inquired whether the compound causes activation of HSF1 DNA-binding activity. EMSA using extracts from HeLa cells exposed to IHSF115 revealed slightly increased HSE probe binding relative to extracts from vehicle-treated cells (Figure 5c). Refreshment of the IHSF115-containing medium after 2 h in cells exposed for 4 h or after 2 h and 4 h in cells exposed for 6 h resulted in considerably higher binding signals. The HSE binding signal was quantitatively supershifted by an HSF1 antibody. To investigate transcriptomic changes, HeLa cells were exposed to 25 µM IHSF115 or vehicle for 6 h (with medium refreshed). Transcript levels were assessed via hybridization to Affymetrix microarrays (data corroborated with RT-qPCR for selected genes (Table S12)). The apparent expression of 240 genes was enhanced at least 1.5-fold in cells exposed to IHSF115 (Table S13). The transcript levels of 134 of these genes were also significantly elevated in heat-stressed cells (data from Ref. [18]). The 60 genes whose transcript levels were elevated the most in IHSF115-exposed cells are listed in Figure 5d. Fold increases caused by IHSF115 and heat, respectively, are shown in the first two columns of the heatmap. It appeared that IHSF115 induced a transcriptomic response that resembled but was not identical to a typical heat stress response. GO analysis of transcripts whose levels were increased in IHSF115- and heat-exposed cells revealed that many of the same processes were highly enriched (Figure 5e and Table S14). The most important differences concerned the categories “chromatin silencing” and “nucleosome assembly”. These differences may be largely explained by increased transcript levels of several histone genes in IHSF115-exposed cells that may have resulted from transcript stabilization rather than enhanced expression (third column in Figure 5d and Table S15).

### 2.5. Elevated Sensitivity of MM Cells to CEL, WA and IHSF

**Figure 6:**
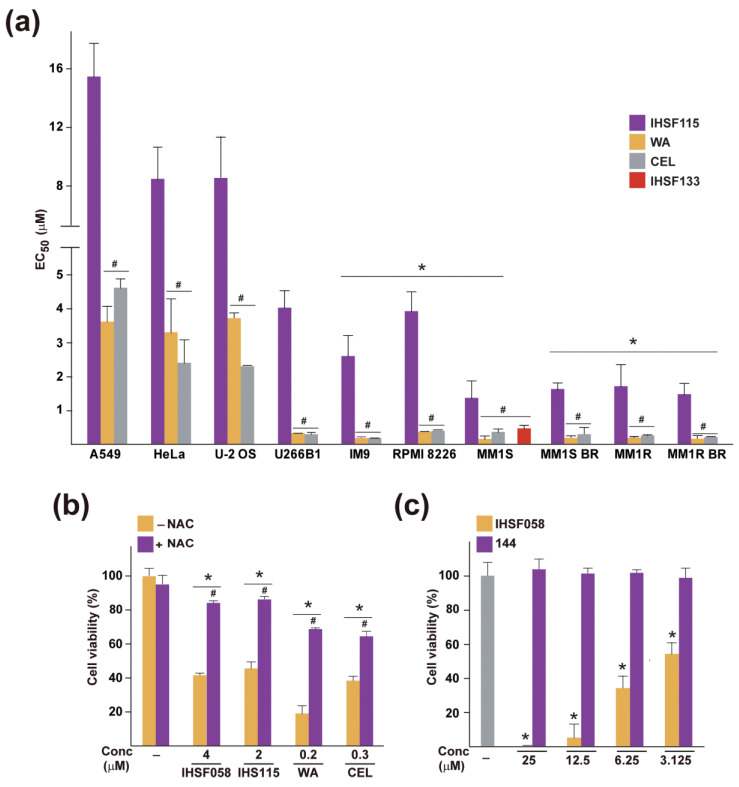
Effects of IHSF, CEL and WA on the viability of MM cell lines and other cancer cell lines. (**a**) EC50 values (50% viability after 96 h of exposure estimated using alamar blue assay) for MM cell lines U266B1, IM9, RPMI 8226, MM1S, MM1R and MM1S BR and MM1R BR cells selected for bortezomib resistance and reference cancer cell lines A549 (lung cancer), HeLa (cervical cancer) and U-2 OS (osteosarcoma). *: *p* < 0.05 (compared to reference cancer cell lines exposed to IHSF115, CEL or WA). #: *p* < 0.05 (compared to the corresponding cell line treated with IHSF115). (**b**) Parallel cultures of MM1S cells were exposed for 96 h to vehicle (-) or the indicated concentrations of compounds in the presence or absence of 0.1 mM NAC. Viability was estimated as in (**a**). *: *p* < 0.05 (compared to cells exposed to vehicle, in the presence or absence of NAC); #: *p* < 0.05 (compared to cells treated with compound in the absence of NAC). (**c**) Parallel cultures of MM1S cells were exposed for 96 h to the indicated concentrations of IHSF058 or compound 144. Viability was estimated as in (**a**). *: *p* < 0.05 (compared to cells exposed to vehicle).

We next inquired whether the common ability of CEL, WA and IHSF115 to induce extensive protein unfolding, which can be inferred to underlie their cytotoxicity, is reflected in a similar differential sensitivity of target cell types towards the compounds. We had previously observed that MM cells are more sensitive to IHSF115 than other cancer cells [18]. Here, we compared the effects of CEL, WA and IHSF115 on viability in a set of MM cell lines (U266B1, IM9, RPMI 8226 and MM1S) and a set of other cancer cell lines (A549, HeLa and U-2 OS). We observed that all MM cell lines were considerably more sensitive to the compounds than the reference cancer cell lines (Figure 6a). The differences in sensitivity were most striking for CEL and WA, which were about an order of magnitude more effective in killing MM cells than the reference cells. Dexamethasone-resistant MM1R cells were as susceptible to the compounds as the steroid-sensitive MM1S cells. The compounds were as effective in MM1S and MM1R cells selected for resistance to 5 nM concentrations of proteasome inhibitor bortezomib (MM1S BR, MM1R BR) as the original bortezomib-sensitive cells. Note that IHSF133, an analog of IHSF115, approached the potency of CEL and WA. We conclude that MM cells are particularly susceptible to killing by CEL, WA and IHSF (in particular, IHSF133). It appears that the α,β-unsaturated carbonyl group present in the compounds is responsible for their cytotoxicity in MM cells. NAC (0.1 mM) essentially neutralized (IHSF058 and IHSF115) or greatly reduced (CEL and WA) the cytotoxicity of the compounds in MM1S cells (Figure 6b), and compound 144 did not cause any loss in viability (Figure 6c).

### 2.6. Synergistic Interactions of CEL, WA and IHSF115 with Proteasome Inhibitor Bortezomib in MM Cells

Both proteasome inhibitors and the compounds of this study interfere with proteostasis. We, therefore, explored whether bortezomib synergistically interacted with CEL, WA and IHSF115. The cytotoxicity of binary combinations of each of the latter compounds and bortezomib were assessed in MM cell lines MM1S, IM9 and RPMI 8226. We found synergism or at least moderate synergism for all combinations, particularly at high effect levels (Table 1).

## 3. Discussion

The most surprising finding of the present study was that compounds IHSF115, IHSF058 and WA caused pervasive unfolding of proteins at concentrations at which they are cytotoxic as estimated by TMT mass-spectrometric analysis of detergent-insoluble extract fractions from compound- and vehicle-treated cells. This analysis revealed that, of the 5132 (commonly) detected cellular proteins, 991 (IHSF115), 915 (WA) and 722 (IHSF058) were enriched at least 1.5-fold in the detergent-insoluble fractions. Hence, the compounds caused significant denaturation of 14–19% of all detected proteins. That CEL provoked an analogous response was evident from WB experiments. The WB experiments also served to confirm the mass-spectrometric observations for IHSF115, IHSF058 and WA. It might be argued that, because the mass spectrometric analyses for IHSF115 and WA had been performed using cells that had been exposed to compound concentrations that produced nearly maximal cytotoxic effects, the observed broad unfolding of proteins may not be causally related to the cytotoxicity of the compounds. This is clearly not the case for the less active compound IHSF058, which was tested at a concentration that was close to its EC50 value for cytotoxicity (18.7 µM). The WB experiments further revealed that all compounds caused detectable unfolding of proteins at 3.125–6.250 µM concentrations. Important fractions of some of the tested proteins, in some cases approaching or even surpassing 50% of total protein amounts, became denatured at higher compound concentrations. Results from additional experiments suggested that the α,β-unsaturated carbonyl group present in the study compounds is responsible for the covalent modification of proteins, for the unfolding of proteins as well as for the cytotoxicity caused by the compounds (Figure 4 and Figure 6). It appears therefore reasonable to propose that the cytotoxicity of IHSF and the natural products is a general consequence of the pervasive and often massive unfolding of proteins the compounds cause. We hypothesize that the functionality of many or most signaling and effector pathways may be significantly compromised by the study compounds. The pathways may underperform or even become dysfunctional. Different cells may respond differently to the compound-induced proteostasis crisis. As an illustration, the main mode of death induced by IHSF115 is apoptotic in MM cell line MM1S but non-apoptotic in HeLa cells [18].

The PN network could be expected to mitigate the damage caused by pervasive covalent modification of proteins. However, salvaging effects of the proteostasis system may be limited since the study compounds also target, directly or indirectly, many constituent proteins of the PN, including chaperones, cochaperones and other proteins involved in the heat shock response; components of the proteasome; proteins of the ubiquitin system; autophagy-related proteins and translation initiation factors (Table S16). Neither covalent modification nor aggregation could be documented for endoplasmic reticulum stress-related proteins (CHOP, IRE1, PERK, ATF6, XBP1, BIP, GADD34, ATF4, EIF2α, S1P and S2P). In a limited set of experiments, we were able to demonstrate that modification and/or aggregation of components of the PN has functional consequences; IHSF115 was found to inhibit all three protease activities of the proteasome in a dose-dependent manner as well as to affect the overall level of ubiquitinated proteins. It may be further noteworthy that of the 601 known ubiquitinated proteins that were detectable in our TMT mass-spectrometric assays, 92, 74 and 63 aggregated in cells exposed to IHSF115, WA and IHSF058, respectively, and 141 were covalently modified by IHSF058 (Table S16. For ubiquitinated proteins in HeLa cells see Ref. [56]).

To rationalize the different pharmacological activities of natural compounds CEL and WA, numerous studies have been carried out to identify targeted proteins [20,21,22,23,24,25,29,30,31,32,33,34,35,38,39,42,43,45,46,47,48,49,50,51,52,53]. Regarding WA, we observed that many of the proteins or members of protein categories that had been reported as targets did in fact aggregate. They included NFKB1, NFKB2, IKKB, NEMO, components of the ubiquitin-proteasome system, autophagy-related proteins, HSF1, chaperones and co-chaperones, AKT1 and AKT2 and translation initiation-related factors (Tables S1–S3 and S16). Not detectably aggregated were the β5-subunit of the 20S proteasome PSB5, β-tubulin (except for TUBB3), HDAC6; HSP90, vimentin (VIME), and annexins A2 and A4.

We found that 1178 proteins from cells that had been exposed to biotinylated IHSF058 were highly enriched by streptavidin beads, indicating that these proteins were covalently modified by the compound (Table S9). Using the same number for reliably detectable proteins as before (5132), this suggests that over 20% of all detectable cellular proteins were targeted directly by the compound. Surprisingly, only 150 of these proteins were found to accumulate in the detergent-insoluble fraction of IHSF058-exposed cells (Table S10). Only 100 of the latter proteins were among the 440 proteins that commonly aggregated in cells exposed to IHSF058, IHSF115 and WA. Hence, 1028 proteins were covalently modified by IHSF058 but did not aggregate to levels that the TMT mass-spectrometric assays were able to detect. Our WB blot experiments revealed that covalently modified proteins abounded in the detergent-soluble fraction (Figure 4f). It seems reasonable to assume that their covalent modification compromised many of these proteins functionally and, perhaps, even structurally. Hence, the 722 proteins whose accumulation in the detergent-insoluble fraction could be demonstrated (Table S2) only revealed the tip of the iceberg. The disruption of proteostasis brought about by IHSF058 (and, by extension, the other study compounds) was likely to be far more important than even the observed pervasive accumulation of aggregated proteins suggested.

**Figure 7:**
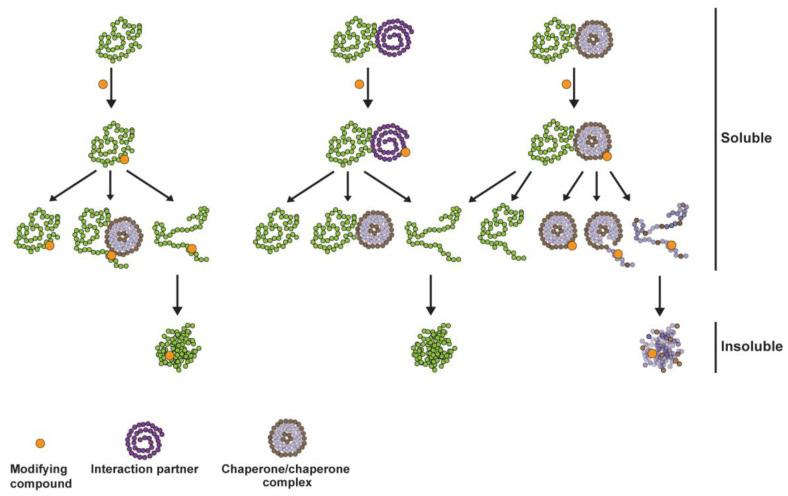
Schematic depiction of events proposed to occur subsequent to compound-induced covalent modification of proteins. Green chains: proteins of interest. Additional proteins that may aggregate include proteins that were to be modified but were not modified as well as proteins that were to be eliminated but were not eliminated because of a failing proteostasis system.

Conversely, of the 722 proteins that were found to aggregate in IHSF058-exposed cells, 572 could not be shown to be covalently modified by IHSF058. We assume that many or most of these proteins unfolded because their conformational stability depended on interactions with proteins that were covalently modified and, as a consequence of their modification, had become unable to support these interactions. Alternatively, or in addition, the accumulation of modified proteins may have overtaxed the already failing cellular chaperoning system, with the result that some unmodified proteins that are dependent on chaperoning also unfolded. These considerations raise the possibility that the proteostasis crisis caused by the compounds may have been even more severe than became apparent from our data. Many more proteins that depended on interactions with other proteins or chaperones than the 572 proteins whose aggregation we were able to document may have been inactivated and/or conformationally altered, albeit not to a degree that led to their substantial aggregation. Figure 7 schematically depicts the suggested consequences of covalent modification of proteins. As a corollary, our study implies that cells exposed to the study compounds may suffer death from multiple failures caused by protein modification, some more important than others depending on cellular background, rather than because of the inactivation of a particular protein or pathway as previously envisaged.

We found that multiple MM cell lines are highly sensitive to CEL, WA and IHSF (Figure 6). EC50 values for CEL, WA and IHSF133 were about an order of magnitude lower for various MM cell lines, including hormone-resistant lines and proteasome inhibitor-resistant cells compared to several other cancer cell lines. Thus, an adequate therapeutic window may exist for the use of these compounds as single agents in the therapy of MM. It is noted that CEL was previously shown to be active in an MM xenograft model [24]. Resistance may be a lesser problem for the study compounds than for other active agents currently employed in MM therapy because of their different mechanisms of action. This difference in mechanisms may also explain why the compounds interact with proteasome inhibitor bortezomib in a synergistic manner (see also Ref. [32]). Combination therapy involving a proteasome inhibitor and one of the study compounds seems worth investigating.

## 4. Materials and Methods

### 4.1. Compounds, PLASMIDS and Viruses

IHSF001 ((E)-ethyl 4-oxo-4-(thiazol-2-ylamino)but-2-enoate) was obtained from AKos Consulting & Solutions GmbH (Klarenbeek, Netherlands). The syntheses of IHSF058-115 were described in Ref. [18] and those of IHSF133 and compounds 142-144, in the Supplementary Materials. WA was sourced from Santa Cruz Biotechnology (Dallas, TX, USA) and CEL, from Merck (Kenilworth, NJ, USA). All compounds were dissolved in DMSO at 10 mM. phRL-CMV and pCMV-Luc were obtained from Promega (Madison, WI, USA) and Origene (Rockville, MD, USA), respectively. pCMV-β-Gal was previously described [57]. Adenovirus Ad-CMV-RLUC was obtained from SignaGen Laboratories (Frederick, MD, USA).

### 4.2. Cell Culture, Cell Viability and Reporter Assays

Cell lines, except HEK293 cells, and culture conditions were described previously [18,58]. HEK293 cells (ATCC CRL-1573) were cultured in Dulbecco’s modified Eagle’s medium (Lonza, Basel, Switzerland) supplemented with 10% fetal bovine serum, 10 U/mL penicillin and 0.01 mg/mL streptomycin. To select bortezomib-resistant cell pools, MM1S and MM1R cells were cultured in RPMI-1640 medium (Lonza) supplemented with 10% fetal bovine serum, 10 U/mL penicillin, 0.01 mg/mL streptomycin and a gradient of increased concentrations of bortezomib (ApexBio Technology, Houston, TX, USA), i.e., 0.1, 0.2, 0.4, 0.6, 0.8, 1, 1.5, 2, 2.5 and 5 nM. Each concentration was maintained for 3-5 passages (2–3 weeks). Treatment of unselected MM1S and MM1R cells with 5 nM bortezomib for 96 h resulted in total loss of viable cells, as determined via trypan blue exclusion assay.

The viability of cells, taken as a proxy for cytotoxicity, was investigated using an alamar blue assay [18]. Interactions between compounds were analyzed using the Calcusyn software.

Transfections employed Lipofectamine 2000 reagent (Invitrogen, Waltham, MA, USA). Renilla luciferase, firefly luciferase, and β-galactosidase activities were determined using the Renilla-Glo Luciferase, One-Glo Luciferase, and Beta-Glo Assay Systems (all from Promega), respectively.

### 4.3. Detergent-Insoluble and Detergent-Soluble Extract Fractions

Cells were extracted with a lysis buffer (20 mM Tris-HCl pH7.4, 10 mM EDTA, 100 mM NaCl and 1% Triton X-100) supplemented with 1 mM phenylmethylsulfonyl fluoride and a protease inhibitor cocktail (Halt; Thermo Fisher Scientific, Waltham, MA, USA). Extracts were centrifuged at 18,500 g for 10 min at 4 °C to obtain detergent-soluble and detergent-insoluble fractions.

### 4.4. Western Blot (WB)

Proteins were resolved via SDS-PAGE, transferred to a PVDF membrane and detected via indicated antibodies. GAPDH served as a loading control. In some experiments, PVDF membranes were incubated with streptavidin conjugated to horseradish peroxidase (Streptavidin-HRP; Thermo Scientific, Waltham, MA, USA).

### 4.5. Tandem Mass Tag (TMT) Mass Spectrometry

Detergent-insoluble extract fractions were digested with modified trypsin (Promega) using the FASP protocol [59]. Equal amounts of each peptide sample were labeled using the 10-plex TMT Reagents (Thermo Fisher Scientific) and then analyzed via liquid chromatography-mass spectrometry (LC-MS/MS) [60,61,62,63].

### 4.6. Analysis of Covalent Protein Modification

Cells were extracted with a lysis buffer (50 mM Tris-HCl pH8.0, 100 mM NaCl and 0.5% IGEPAL CA-630) supplemented with the above-mentioned protease inhibitors. Extracts were incubated for 1 h at 4 °C with streptavidin-coated magnetic particles (Roche, Basel, Switzerland). Bound proteins were digested using the FASP protocol and then analyzed via LC-MS/MS.

### 4.7. Electrophoretic Mobility Shift Assay (EMSA), RT-qPCR and Transcriptomic Assays

A heat shock element (HSE) probe fragment labeled with [α-32P]dCTP was used to perform EMSA assays [18,64,65,66].

Total RNA was extracted with an RNeasy Mini Kit (Qiagen, Hilden, Germany). To quantify HSPA1A mRNA, cDNA was prepared from total RNA using Transcriptor reverse transcriptase and an anchored oligo (dT)18 primer (both from Roche). qPCR was performed employing LightCycler FastStart DNA Master SYBR Green I (Roche) and oligonucleotides 5′-ACAGGCTGGTGAACCACTTC-3′ (F) and 5′-CCCTGGTGATGGACGTGTAG-3′ (R).

For transcriptomic analysis, total RNA was processed using a GeneChip WT PLUS Reagent kit and hybridized with GeneChip Human Gene 2.0 ST Array (both from Applied Biosystems, Waltham, MA, USA). Data were corroborated using RT-qPCR for selected genes. cDNA was prepared from total RNA employing a High-Capacity RNA-to-cDNA Kit, and qPCR was performed using TaqMan Gene Expression Assays (both from Life Technologies, Carlsbad, CA, USA). Gene ontology analyses were based on DAVID [67].

### 4.8. Statistics

Unless indicated otherwise, data are presented as means or means ± SD, of at least three independent experiments performed in triplicate. Data were tested for normality using the Shapiro–Wilk test. All normally distributed data were analyzed using Student’s *t*-test or one-way ANOVA followed by Dunett’s multiple comparisons tests. For data not normally distributed, statistical analyses were performed using the non-parametric Kruskal-Wallis test followed by Dunn’s multiple comparisons tests. All analyses were performed using GraphPad Prism Version 7.00. Microarray data were analyzed using the limma software included in Transcriptome Analysis Console (Applied Biosystems). The criterion for significance in statistical analyses was set at *p* ≤ 0.05.

## Figures and Tables

**Table 1:** Interactions of CEL, WA and IHSF115 with bortezomib in MM cells.

Compound	Cell Line	Ratio Compound/Bortezomib	Combination Index Values (CI) at Different Effect Levels (ED) *		
			ED_50_	ED_75_	ED_90_
Celastrol	MM1S	200	0.60	0.60	**0.60**
		160	0.74	0.67	**0.14**
	IM9	75	1.12	0.95	**0.81**
		67	0.77	0.56	**0.42**
	RPMI 8226	100	1.29	0.77	**0.50**
		100	1.25	0.98	**0.77**
Withaferin A	MM1S	50	1.20	0.86	**0.63**
		40	1.38	0.99	**0.75**
	IM9	40	1.08	0.82	**0.66**
		33	0.99	0.79	**0.67**
	RPMI 8226	175	0.95	0.56	**0.34**
		150	0.80	0.50	**0.31**
IHSF115	MM1S	1200	1.33	1.00	**0.78**
		1300	1.26	0.95	**0.73**
	IM9	700	1.29	0.91	**0.65**
		600	1.25	0.98	**0.78**
	RPMI 8226	1000	1.40	0.99	**0.71**
		800	1.63	1.16	**0.86**

* CI range of 0.3–0.7: synergism; 0.7–0.85: moderate synergism; 0.85–0.9: slight synergism; 0.9–1.10: near additivity; 1.10–1.20: slight antagonism; 1.20–1.45: moderate antagonism.

## Data Availability

Openly available gene expression data were deposited at www.ncbi.nlm.nih.gov/geo/query/acc.cgi?acc=GSE209688, available to public on 28 July 2022. Other data presented in this study are available on reasonable request from the corresponding authors.

## Supplementary Materials

The following supporting information can be downloaded at https://www.mdpi.com/article/10.3390/ph16040616/s1: Supplementary Methods, Contents of Supplementary Tables S1–S16 and Supplementary Tables S1–S16.
