# Supplementary material for: Disruption of Proteostasis by Natural Products and Synthetic Compounds That Induce Pervasive Unfolding of Proteins: Therapeutic Implications
Source: Pharmaceuticals (Basel). 2023 Apr 19;16(4):616. doi: 10.3390/ph16040616 (PMC10145903; doi:10.3390/ph16040616)
Supplement: Supplementary file 1 [file pharmaceuticals-16-00616-s001.zip › Contents of Supplementary Tables S1-16.pdf]

Contents of Supplementary Tables S1-16

Table S1. Accumulation of proteins in insoluble extract fractions of IHSF115-treated cells as determined by TMT mass spectrometry. Nine samples from vehicle-treated cells and 5 samples from IHSF115-treated cells were analyzed in 4 independent experiments. Zq: standardized log2 protein levels; C: vehicle-treated; Np: number of peptides detected. Exp.: experiment. The differential coloring in the ΔZq (IHSF115 - C) column relates to relative levels of accumulation.

Table S2. Accumulation of proteins in insoluble extract fractions of IHSF058-treated cells. 9 samples from vehicle-treated cells and 3 samples from IHSF058-treated cells were analyzed in 4 independent experiments. For other details, see Table S1.

Table S3. Accumulation of proteins in insoluble extract fractions of WA-treated cells. 9 samples from vehicle-treated cells and 3 samples from WA-treated cells were analyzed in 4 independent experiments. For other details, see Table S1.

Table S4. Commonly unfolding proteins in IHSF115-, IHSF058- or WA- treated cells. For other details, see Table S1.

Table S5. Gene ontology analyses of the commonly unfolding proteins.

Table S6. Rank order of the 100 most overrepresented proteins in insoluble extract fractions of IHSF115-treated cells. For comparison, the rank orders of the same proteins in insoluble extract fractions of IHSF058- or WA- treated cells are also provided. For other details, see Table S1.

Table S7. Rank order of the 100 most overrepresented proteins in insoluble extract fractions of IHSF058-treated cells. For comparison, the rank orders of the same proteins in insoluble extract fractions of IHSF115- or WA- treated cells are also provided. For other details, see Table S1.

Table S8. Rank order of the 100 most overrepresented proteins in insoluble extract fractions of WA-treated cells. For comparison, the rank orders of the same proteins in insoluble extract fractions of IHSF115- or IHSF058-treated cells are also provided. For other details, see Table S1.

Table S9. Proteins covalently modified by compound 143. Samples from four independent experiments were analyzed in duplicate. Biotinylated proteins in cells exposed to compounds 142 or 143 were isolated using streptavidin-coated magnetic particles and identified by liquid chromatography-mass spectrometry.

Table S10. List of proteins that accumulated in the insoluble extract fraction of IHSF058-treated cells as well as were covalently modified by compound 143.

Table S11. Gene ontology analysis of the proteins listed in Table S10.

Table S12. Effects of IHSF115 on transcript levels of selected genes in HeLa cells examined by hybridization to Affymetrix microarrays and by RT-qPCR analysis. In the IHSF115/C and (IHSF115 + ActD)/ActD columns, red colors denote significantly increased transcript levels in IHSF115-treated cells and green colors significantly reduced transcript levels. C: vehicle; ActD: actinomycin D.

Table S13. Transcriptomic analysis of HeLa cells treated with IHSF115 or vehicle. Three independent experiments were performed. Column IHSF115/C relates to ratios of transcript levels in IHSF115- and vehicle-treated cells. Column H/C relates to ratios of transcript levels in heat-treated (H) and vehicle-treated (C) HeLa cells (data imported from ref.18). In the IHSF115/C and H/C columns, red colors denote significantly increased transcript levels in IHSF115- or heat-treated cells and green colors significantly reduced transcript levels. FDR: false discovery rate.

Table S14. Gene ontology analyses of genes whose transcript levels were significantly increased in IHSF115- and heat-treated cells.

Table S15. Transcriptomic analysis of HeLa cells treated with IHSF115/Act D or with Act D. Two independent experiments were performed. Column (IHSF115 + ActD)/ActD relates to ratios of transcript levels in IHSF115/Act D- and Act D-treated cells. Column IHSF115/C relates to ratios of transcript levels in IHSF115- and vehicle-treated cells. For other details see Table S13.

Table S16. PN components and proteins known to be ubiquitinated that are targeted by IHSF058, IHSF115 and WA. This compilation is based on data reported in Tables S1-4 and S9.
