# Supplementary material for: Disruption of Proteostasis by Natural Products and Synthetic Compounds That Induce Pervasive Unfolding of Proteins: Therapeutic Implications
Source: Pharmaceuticals (Basel). 2023 Apr 19;16(4):616. doi: 10.3390/ph16040616 (PMC10145903; doi:10.3390/ph16040616)
Supplement: Supplementary file 1 [file pharmaceuticals-16-00616-s001.zip › Supplementary Methods.pdf]

Supplementary Methods

Compounds, plasmids and viruses

IHSF001 ((*E*)-ethyl 4-oxo-4-(thiazol-2-ylamino)but-2-enoate) was obtained from AKos Consulting & Solutions GmbH (Klarenbeek, Neherlands). The syntheses of IHSF058, 070, 090 and 115 were described previously (I). phRL-CMV and pCMV-Luc were obtained from Promega, Madison, WI) and Origene (Rockville, MD), respectively. pCMV-βGal was previously described (II). Adenovirus Ad-CMV-RLUC was from SignaGen Laboratories (Frederick, MD).

Synthesis of IHSF133 and compounds 142-144

IHSF133

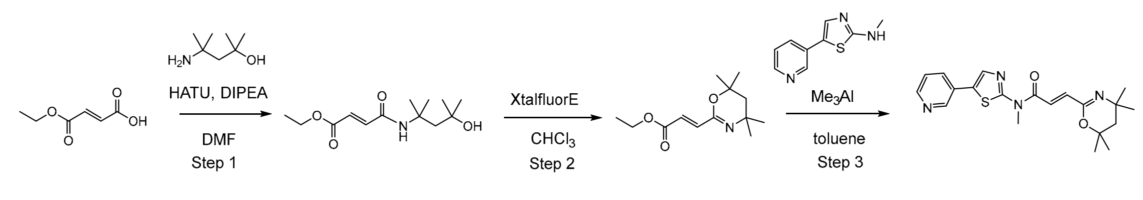

Step 1: HATU (1.3g, 3.47mmol) was added to a mixture of ethyl hydrogen fumarate (500mg, 3.47mmol), 4-amino-2,4-dimethyl-pentan-2-ol (546mg, 4.17mmol) and DIPEA (1.81ml, 10.4mmol) dissolved in DMF (10ml). The reaction mixture was stirred at room temperature for 2h and then diluted with ethyl acetate (50ml), and washed with water (2x30ml) and brine (30ml). The organic fraction was passed through a phase separator cartridge (Biotage, Uppsala, Sweden), was evaporated and purified by flash column chromatography (isohexane -> EtOAc) to afford ethyl (E)-4-((4-hydroxy-2,4-dimethylpentan-2-yl)amino)-4-oxobut-2-enoate (700mg, 78%) as a yellow oil.

Step 2: Ethyl (*E*)-4-((4-hydroxy-2,4-dimethylpentan-2-yl)amino)-4-oxobut-2-enoate (350mg, 1.36mmol) and Xtalfluor-E (374mg, 1.63mmol) were dissolved in chloroform (10ml). The reaction mixture was sealed and heated to 90^{0}C for 3h and then allowed to cool. The solvent was evaporated and the crude purified by flash column chromatography (isohexane -> EtOAC). A second purification was required (DCM-> 10%MeOH/DCM). The material was then loaded onto an SCX-2 column (Biotage, 5g). The column was washed several times with MeOH, then the bound material was eluted with NH3/MeOH. The solvent was evaporated to afford ethyl (E)-3-(4,4,6,6-tetramethyl-5,6-dihydro-4H-1,3-oxazin-2-yl)acrylate (123mg, 38%) as a yellow oil.

Step 3: *N*-methyl-5-(pyridine-3-yl)triazol-2-amine (117mg, 0.62mmol) was dissolved in toluene (3ml). The reaction mixture was cooled to 0^{0}C and trimethylaluminium (0.51ml, 2M in toluene, 1.03mmol) was added under nitrogen. The reaction mixture was stirred at room

temperature for 30min, before adding ethyl (E)-3-(4,4,6,6-tetramethyl-5,6-dihydro-4H-1,3-oxazin-2-yl)acrylate (123mg, 0.51mmol) dissolved in toluene (1ml). The mixture was then heated to 90^{0}C for 4h. A solution of Rochelle's salt (10ml) was added, and the mixture was stirred for 30min before diluting with water and 2M aq NaOH. The reaction mixture was extracted with DCM (2x20ml), and the organic layers were combined, passed through a phase separator cartridge (Biotage) and evaporated. The material was successfully purified by reverse phase prep-HPLC (XBridge (Northhampton, UK) C18 OBD 19x200 mm, MeCN-water, pH9). The fractions containing the desired target material were combined and were extracted into DCM. The solvent was evaporated and the material dried in a desiccator overnight to afford (*E*)-N-methyl-N-(5-(pyridin-3-yl)thiazol-2-yl)-3-(4,4,6,6-tetramethyl-5,6-dihydro-4H-1,3-oxazin-2-yl)acrylamide (3.5mg, 2%) as a yellow solid (LCMS (pH9): 8.05min; m/z=385.2 $[M+H]^+$; 97.02%; LCMS (pH2): 4.73min; m/z=385.2 $[M+H]^+$; 98.08%; ^{1}H-NMR (400MHz, DMSO-d6): δ 8.72 (d, J=1.4Hz, 1H), 8.34 (dd, J=4.6Hz, 1.4Hz, 1H), 7.95 (s, 1H), 7.86-7.91 (m, 1H), 7.28 (dd, J=8.2Hz, 4.6Hz, 1H), 7.1 (d, J=15.1Hz, 1H), 6.67 (d, J=15.1Hz, 1H), 3.62 (s, 3H), 1.65 (s, 2H), 1.19 (s, 6H), 1.07 (s, 6H)).

Compound 142

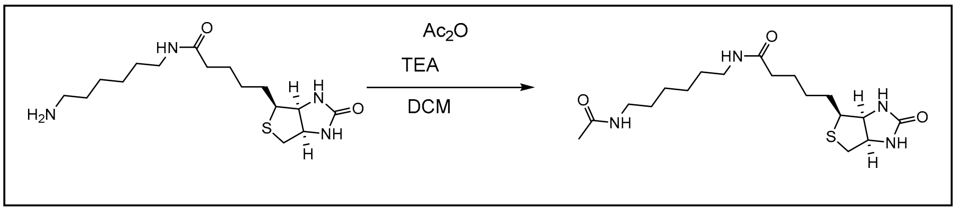

N-biotinyl-1,6-hexane diamine (50mg, 0.143mmol) was suspended in DCM (1ml) and triethylamine (0.07ml, 0.429mmol). Acetic anhydride (0.02ml, 0.172mmol) was added and the reaction mixture was stirred at room temperature for 3h. The reaction mixture was quenched with MeOH and was evaporated to dryness. The crude material was suspended in MeOH and was loaded onto an SCX-2 column (Biotage). The column was washed several times with MeOH, then the bound material was eluted with NH_3/MeOH. The solvent was evaporated and the material dried in a desiccator overnight to afford *N*-(6-acetamidohexyl)-5-((3aS,4S,6aR)-2-oxohexahydro-1H-thieno[3,4-d]imidazol-4-yl)pentanamide (31mg, 55%) as a white solid (LCMS (pH9): 5.55min; m/z=385.3 $[M+H]^+$; 99.21% by ELSD; LCMS (pH 2): 5.58min; m/z=385.4 $[M+H]^+$; 100% by ELSD; ^{1}H-NMR (400MHz, DMSO-d6): δ 7.79 (t, J=5.0Hz, 1H), 7.74 (t, J=5.5Hz, 1H), 6.44 (s, 1H), 6.37 (s, 1H), 4.27-4.34 (m, 1H), 4.10-4.16 (m, 1H), 3.06-3.13 (m, 1H), 2.95-3.05 (quint, J=6.4Hz , 4H), 2.82 (dd, J=12.8Hz, 5.0Hz, 1H), 2.58 (d, J=12.4Hz, 1H), 2.05 (t, J=7.3Hz, 2H), 1.78 (s, 3H), 1.18-1.68 (m, 14H)).

Compound 143

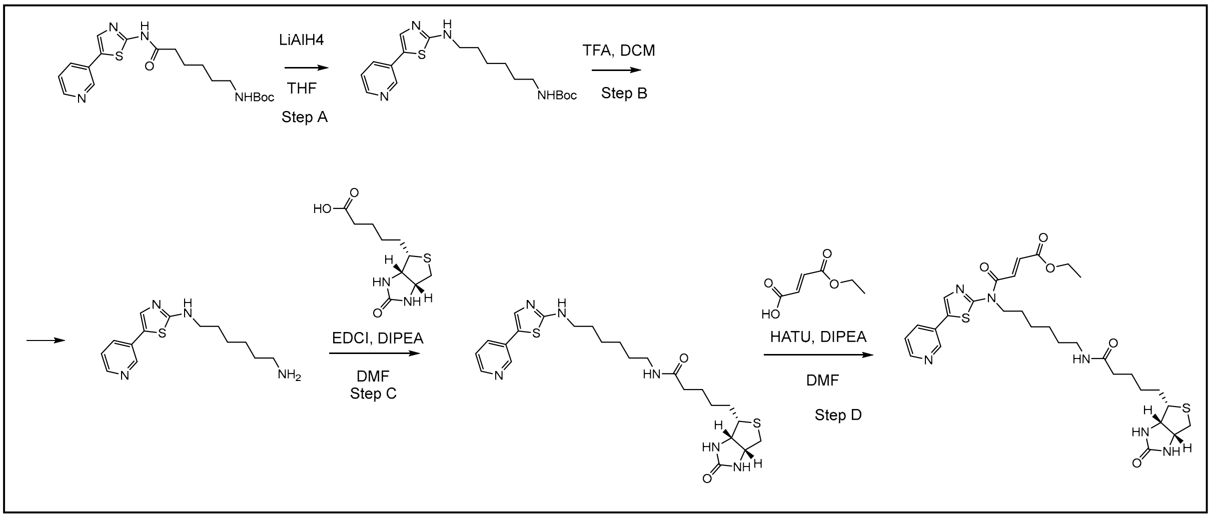

Step A: tert-butyl (6-oxo-6-((5-(pyridin-3-yl)thiazol-2-yl)amino)hexyl)carbamate (400mg, 1.024mmol) was dissolved in dry THF (50 ml) and LiAlH4 (1M in THF, 5.1ml, 5.12mmol) was added dropwise at 0^0C. The reaction mixture was allowed to slowly warm to room temperature and was stirred overnight. The reaction mixture was quenched with water (5ml) and 2M aq NaOH (10ml). The suspension was extracted with EtOAc, the organic layer was washed with 2M aq NaOH, water then brine, and was passed through a phase separator cartridge (Biotage). The solvent was evaporated, and the residue was purified by flash column chromatography (isohexane -> 10 % EtOAc/isohexane) to afford tert-butyl (6-((5-(pyridin-3-yl)thiazol-2-yl)amino)hexyl)carbamate (120mg, 31%).

Step B: tert-butyl (6-((5-(pyridin-3-yl)thiazol-2-yl)amino)hexyl)carbamate (120mg, 0.319mmol) was dissolved in DCM (1ml) and TFA (1ml) was added. The reaction mixture was stirred at room temperature for 1h. The solvent was evaporated to afford N1-(5-(pyridin-3-yl)thiazol-2-yl)hexane-1,6-diamine hydrochloride (130mg, 100%) as an orange/yellow oil. This was used in the next step without purification.

Step C: N1-(5-(pyridin-3-yl)thiazol-2-yl)hexane-1,6-diamine hydrochloride (130mg, 0.415mmol), 5-((3aS,4S,6aR)-2-oxohexahydro-1H-thieno[3,4-d]imidazol-4-yl)pentanoic acid (110mg, 0.485mmol), 1-ethyl-3-(3-dimethylaminopropyl)carbodiimide (200mg, 1.04mmol) and DIPEA (0.57ml, 3.25mmol) were dissolved in DMF (2ml) and stirred at room temperature overnight. The reaction mixture was partitioned between EtOAc and water. The organic layer was evaporated and purified by flash column chromatography (DCM-> 20%MeOH/DCM) to afford 5-((3aS,4S,6aR)-2-oxohexahydro-1H-thieno[3,4-d]imidazol-4-yl)-N-(6-((5-(pyridin-3-yl)thiazol-2-yl)amino)hexyl)pentanamide (180mg, 86%) as a yellow solid.

Step D: 5-((3aS,4S,6aR)-2-oxohexahydro-1H-thieno[3,4-d]imidazol-4-yl)-N-(6-((5-(pyridin-3-yl)thiazol-2-yl)amino)hexyl)pentanamide (80mg, 0.159mmol) and (E)-4-ethoxy-4-oxobut-2-enoic acid (6 mg, 0.425mmol) were dissolved in a mixture of DMF (2ml) and DIPEA (0.56ml, 0.425mmol). HATU (121mg, 0.319mmol) was added, and the reaction mixture was stirred at room temperature overnight. The solvent was evaporated and the residue was purified by preparative HPLC to afford ethyl (*E*)-4-oxo-4-((6-(5-((3aS,4S,6aR)-2-oxohexahydro-1H-thieno[3,4-d]imidazol-4-yl)pentanamido)hexyl)(5-(pyridin-3-yl)thiazol-2-yl)amino)but-2-enoate (7mg, 9%) as a yellow solid (LCMS (pH9): 8.11min; m/z=629.4 $[M+H]^+$; 99.11%; LCMS (pH2): 8.08min; m/z=629.4 $[M+H]^+$; 98.92%; ^{1}H-NMR (400MHz, DMSO-d6): δ 8.55 (d, J=4.1Hz, 1H), 8.30 (s, 1H), 7.95 (d, J=7.8Hz, 1H), 7.85 (td, J=7.8Hz, 1.4Hz, 1H), 7.74 (t, J=5.5Hz, 1H), 7.63 (d, J=15.6Hz, 1H), 7.29 (dd, J=7.3Hz, 5.5Hz, 1H), 6.89 (d, J=15.1Hz, 1H), 6.43 (s, 1H), 6.37 (br s, 1H), 4.21-4.38 (m, 5H), 4.11 (dd, J=7.3Hz, 4.5Hz, 1H), 2.95-3.12 (m, 3H), 2.80 (dd, J=12.4Hz, 5.0Hz, 1H), 2.04 (t, J=7.3Hz, 2H), 1.65-1.80 (m, 2H), 1.20-1.65 (m, 16H)).

Compound 144

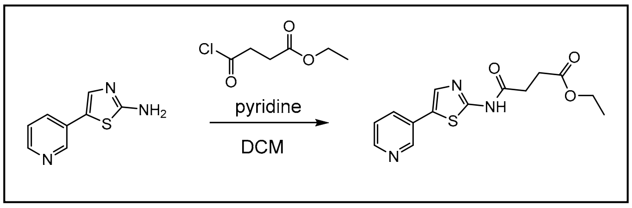

Ethyl 4-chloro-4-oxyutyrate (0.08ml, 0.564mmol) was added to a stirred suspension of 5-(pyridine-3-yl)-1,3-thiazol-2-amine (50mg, 0.282mmol) in DCM (2ml), followed by pyridine (0.114ml, 1.41mmol). The resulting mixture was stirred at room temperature overnight, diluted with water (20ml) and extracted with DCM (20ml). The organic layer was passed through a phase separator cartridge (Biotage), evaporated and purified by preparative HPLC to afford ethyl 4-oxo-4-((5-(pyridin-3-yl)thiazol-2-yl)amino)butanoate (61mg, 71%) as a white solid (LCMS (pH9): 6.90min; m/z=306.2 $[M+H]^+$; 100%; LCMS (pH2): 6.10min; m/z=306.2 $[M+H]^+$; 100%; ^{1}H-NMR (400MHz, DMSO-d6): δ 12.36 (s, 1H), 8.86 (d, J=1.8Hz, 1H), 8.49 (dd, J=5.0Hz, 1.8 Hz, 1H), 7.96-8.04 (m, 2H), 7.44 (dd, J=7.8Hz, 5.0Hz, 1H) , 4.07 (q, J=7.3Hz, 2H), 2.61-2.79 (m, 4H), 1.19 (t, J=7.3 Hz, 3H)).

General abbreviations

Aq., aqueous; DCM, dichloromethane; DIPEA, *N,N*-diisopropylethylamine; DMF, dimethylformamide; DMSO, Dimethyl sulfoxide; ELSD, evaporative light scattering detection; EtOAc, ethyl acetate; HATU, 2-(7-aza-1H-benzotriazole-1-yl)-1,1,3,3-tetramethyluronium hexafluorophosphate; HPLC, high performance liquid chromatography; LCMS, liquid chromatography mass spectrometry; MeOH, methanol; NMR, nuclear magnetic resonance; TFA, trifluoroacetic acid; THF, tetrahydrofuran.

Cell culture, cell viability and reporter assays

Cell lines, except HEK 293, and culture conditions were described previously (I, III). HEK 293 (ATCC CRL-1573) cells were cultured in Dulbecco's modified Eagle's medium (Lonza, Basel, Switzerland) supplemented with 10%(v/v) fetal bovine serum, 10U/ml penicillin and 0.01mg/ml streptomycin. Two MM cell lines, MM1S and MM1R, were cultured in RPMI-1640 medium (Lonza) supplemented with 10% fetal bovine serum, 10U/ml penicillin, 0.01mg/ml streptomycin and a gradient of increased concentrations of bortezomib (ApexBio Technology, Houston, TX), i.e., 0.1, 0.2, 0.4, 0.6, 0.8, 1, 1.5, 2, 2.5 and 5nM. Each concentration was maintained for 3-5 passages (2-3 weeks). MM1S and MM1R cells adapted to 5nM bortezomib were named MM1S BR and MM1R BR, respectively.

To investigate cell viability, adherent cells (typically 2×10^4) were seeded in 48-well plates, cultured for 24h and then exposed for 96h to vehicle, compounds of the IHSF series, withaferin A (WA, Santa Cruz Biotechnology, Dallas, TX) or celastrol (CEL; Merck, Kenilworth, NJ). Suspension cells were seeded in 24-well plates at a density of 2×10^5 cells/ml and then exposed for 96h to vehicle or compounds. Medium of adherent cultures was removed, and attached cells were incubated in culture medium containing 10%(v/v) alamar blue dye (Invitrogen, Waltham, MA) for 4h at 37ºC. To investigate the viability of suspension cells, 10%(v/v) alamar blue dye was added directly to the cultures. Medium was collected and, after laser excitation at 530nm, emitted fluorescence at 590nm was quantified using a BioTek Synergy4 multimode plate reader (Agilent Biotechnologies, Santa Clara, CA) (I). To test the interactions of bortezomib with CEL, WA or IHSF115, suspension cells were treated for 48h with compound combinations at constant concentration ratios. Interactions between compounds were analyzed using the software of Calcusyn (Biosoft, Cambridge, UK).

Cells were transfected with phRL-CMV, pCMV-Luc or pCMV-βGal in 96-well plates by using Lipofectamine 2000 reagent (Invitrogen), following the rapid protocol. Briefly, 25 000 cells were transfected with 0.25µl of Lipofectamine 2000, 25ng of reporter plasmid and 135ng of a pBluescript construct. One day after, cells were treated with vehicle or compounds for up to 6h. In some experiments, 10^4 M1 cells, stably expressing *Renilla* luciferase, were seeded in 96-well plates, cultured for 24h and then treated with vehicle or compounds. Cycloheximide (CHX; Merck) was added at 50µg/ml and MG-132 (Merck) at 10µM ten min prior to exposure to compounds or vehicle. In some experiments, cells were treated with vehicle and compounds in the presence of 15mM N-acetylcysteine (NAC; Merck). MM1S cells at a density of 10^6 cells/ml were transduced with Ad-CMV-RLUC at multiplicity of infection of 200. Two h later, cell density was adjusted to 2.5×10^5 cells/ml with fresh medium, and cells were cultured for 24h. *Renilla* luciferase, firefly luciferase and β-galactosidase activities were determined using the Renilla-Glo Luciferase, One-Glo Luciferase and Beta-Glo Assay Systems (all from Promega), respectively.

Detergent-insoluble and detergent-soluble extract fractions

M1 cells were extracted with a lysis buffer (20mM Tris-HCl pH7.4, 10mM ethylenediaminetetraacetic acid (EDTA), 100mM NaCl, 1% Triton X-100) supplemented with 1mM phenylmethylsulfonyl fluoride and a protease inhibitor cocktail (Halt; Thermo Fisher Scientific, Waltham, MA). Cells were lysed by three cycles of rapid freezing (in a dry ice-ethanol bath) and thawing (in a 37^0C water bath). Extracts were incubated on ice for 20min and then centrifuged at 18 500g for 10min at 4°C to obtain detergent-soluble and -insoluble fractions. Protein concentration was determined by a Bradford-based protein assay (Bio-Rad Laboratories, Hercules, CA).

Western blot (WB)

M1 cells (0.6×10^6) were seeded in 60 mm dishes and cultured for 24h. At that time, the cells were pre-exposed for 10min to 50µg/ml CHX and then treated with vehicle or the indicated doses of IHSF058, IHSF115, WA or CEL for 6h. Extracts were prepared and separated into detergent-soluble and detergent-insoluble fractions. Proteins (25µg per lane) were resolved by SDS-PAGE, transferred to a PVDF membrane and detected using the following antibodies: rabbit anti-RLUC polyclonal antibody (PM047; MBL International, Woburn, MA, USA), rabbit anti-human HSF1 polyclonal antibody (ADI-SPA-901; Enzo Biochem, Farmingdale, NY); rabbit anti-human GCLM polyclonal antibody (PA5-92388; Invitrogen), rabbit anti-human STAT3 polyclonal antibodies (PA5-85199; Invitrogen), mouse anti-human HSP27 monoclonal antibody (F-4) (sc-13132; Santa Cruz Biotechnology), mouse anti-human PCNA monoclonal antibody (PC10) (sc-56; Santa Cruz Biotechnology) and mouse anti-human IF4A1/2 monoclonal antibody (H-5) (sc-377315; Santa Cruz Biotechnology). The secondary antibodies used were a goat anti-rabbit antibody (NEF812001EA; PerkinElmer, Waltham, MA) or a rabbit anti-mouse antibody (P0260; Agilent), both conjugated with horseradish peroxidase. The membranes were developed with an enhanced chemiluminescence detection reagent (SuperSignal West Pico PLUS Chemiluminiscent Substrate; Thermo Fisher Scientific).

In one type of experiment, M1 cells, seeded as above, were exposed to 50µg/ml CHX for 10min. Thereafter, 25µM IHSF058, 25µM compound 144 or 15mM NAC, or to vehicle were added to the cultures. Three h later, 25µM compound 143, 25µM compound 142 or vehicle were added, and the cultures were incubated for another 6h. Extracts were prepared and separated into detergent-soluble and detergent-insoluble fractions. Proteins (3µg per lane) were resolved by SDS-PAGE, transferred to a PVDF membrane and incubated with streptavidin conjugated to horseradish peroxidase (Streptavidin-HRP; Thermo Fisher Scientific). The membranes were developed as described above.

A mouse anti-human GAPDH monoclonal antibody (0411) (sc-47724; Santa Cruz Biotechnology) was used for the GPDH loading control.

Tandem mass tag (TMT) mass spectrometry

M1 cells (4.4 ×10^6) were seeded in 150mm dishes and cultured for 24h. At that time, the cells were pre-exposed for 10min to 50µg/ml CHX and then for 6h to 25µM IHSF058, 25µM IHSF115, 12.5µM WA or vehicle. Cells were harvested and detergent-insoluble fractions were prepared. Protein extracts were subjected to in-filter digestion using a filter-aided sample preparation (FASP) protocol (IV). Briefly, extracts were diluted in buffer UA (7M urea in 50mM Tris-HCl pH8.5) and 5mM Tris(2-carboxyethyl) phosphine hydrochloride (TCEP; Thermo Fisher Scientific Pierce) and incubated at room temperature for 15min. Protein alkylation was performed by incubation with 20mM iodoacetamide (Merck) for 20min in the dark. Proteins were loaded onto 10kDa centrifugal filter devices (NanoSep 10k Omega; Pall, New York, NY), and buffer was replaced by washing the filters 3 times with UA buffer and 3 additional times with 50mM ammonium bicarbonate. Proteins were digested overnight at 37°C with modified trypsin (Promega) at a 30:1 protein:trypsin (wt/wt) ratio in 50mM ammonium bicarbonate. The resulting peptides were eluted from the filters with 50mM ammonium bicarbonate and 0.5M NaCl. TFA was added to a final concentration of 1%, and the peptides were desalted on C18 Oasis-HLB cartridges (Waters) and dried.

For stable isobaric labelling, tryptic peptides were dissolved in 100mM triethylammonium bicarbonate buffer, and peptide concentrations were determined by measuring amide bonds using the Direct Detect system (Millipore, Burlington, MA). Equal amounts of peptide samples were labelled using the 10-plex TMT Reagents (Thermo Fisher Scientific) reconstituted with 70µl of acetonitrile according to the manufacturer's protocol. After incubation at room temperature for 1h, reactions were stopped with 0.5% TFA, incubated for 10min and then combined. Samples were concentrated in a Speed Vac, desalted on C18 Oasis-HLB cartridges and then dried. To increase proteome coverage, TMT-labeled samples were fractionated by high-pH reverse phase chromatography using the High pH Reversed-Phase Peptide Fractionation Kit (Thermo Fisher Scientific Pierce) and then dried.

Labelled peptides were analyzed by liquid chromatography-mass spectrometry (LC-MS/MS) using a C-18 reversed phase nano-column (75µm I.D. x 50cm, 2µm particle size, Acclaim PepMap RSLC, 100 C18; Thermo Fisher Scientific) applying continuous acetonitrile gradients from 0-30% B for 360min and 50-90% B for 3min (A= 0.1% formic acid; B=90% acetonitrile, 0.1% formic acid) at 50ºC. Peptides were eluted at a flow rate of 200nl/min from the nano-column to an emitter nanospray needle for real time ionization and peptide fragmentation on a Q Exactive HF Orbitrap mass spectrometer (Thermo Fisher Scientific). An enhanced Fourier transform-resolution spectrum (resolution=70 000) followed by the MS/MS spectra from the 15 most intense parent ions were analyzed along the chromatographic run. Dynamic exclusion was set at 40s.

For peptide identification, all spectra were analyzed with Proteome Discoverer (version 2.1.0.81, Thermo Fisher Scientific) using SEQUEST-HT (Thermo Fisher Scientific). For database searching of the Uniprot database containing all sequences from human and frequently observed contaminants (UniProt_April27_2016_contam; 48,644 entries), parameters were selected as follows: trypsin digestion with 2 maximum missed cleavage sites, precursor and fragment mass tolerances of 2 Da and 0.02 Da, respectively, carbamidomethyl cysteine and TMT modifications at N-terminal and Lys residues as fixed modifications, and methionine oxidation as dynamic modification.

Peptide identification was performed using the probability ratio method (V), and false discovery rate (FDR) was calculated using inverted databases and « the refined method » (VI) with an additional filtering for a precursor mass tolerance of 15ppm (VII). Identified peptides had an FDR equal or lower than 1%. Only those peptides were used to quantify the relative abundance of each protein from reporter ion intensities, and statistical analysis of quantitative data was performed using the WSPP statistical model previously described (VIII). In this model, protein log2-ratios are expressed as standardized variables, i.e., in units of standard deviation according to their estimated variances (Zq values). Proteins with significant differences of Zq values between samples treated with compound and vehicle (ΔZq) of ≥0.5849, corresponding to ≥1.500-fold changes, were included for further analysis.

Analysis of covalent protein modification

M1 cells (4.4×10^{6}) cells were seeded in 150mm dishes and cultured for 24h. At that time, the cells were exposed for 10min to 50µg/ml CHX and then for 6 h to 25µM compound 142 or 25µM compound 143. Cells were extracted with a lysis buffer (50mM Tris-HCl pH8.0, 100mM NaCl, 0.5% IGEPAL CA-630) supplemented with the protease inhibitors described above. Cells were lysed by three cycles of rapid freezing and thawing. Extracts were incubated on ice for 20min, and protein concentrations in unfractioned extracts were determined by a Bradford-based protein assay. Extracts were incubated for 1h at 4°C with streptavidin-coated magnetic particles (Roche, Basel, Switzerland). Particles were washed 3 times with lysis buffer and an additional 4 times with the same buffer lacking IGEPAL CA-630. Beads were then boiled for 5min at 95˚C in a solution containing 50mM Tris-HCl pH8.0 and 4% SDS.

Recovered proteins were diluted in UA buffer containing 50mM dithiothreitol and digested using the FASP protocol as described above. Tryptic peptides were dissolved in 0.2% formic acid and 2% acetonitrile, and loaded on a LC-MS/MS system for online desalting on a 2cm C18 packed pre-column (Thermo Acclaim PepMap 100). Analytical separation was performed on a 50cm column (Thermo Acclaim PepMap 100, 75µm ID × 500mm) equilibrated to 2% buffer B (0.1% formic acid/95% acetonitrile) (20min) by applying a first gradient of 8-31% B (120min), a second gradient of 32-90% B (3min) and 90% B (5min), using an EASY-nLC 1000 HPLC System (Thermo Fisher Scientific). Peptides were eluted

at a flow rate of 200nl/min from the nanocolumn to an emitter nanospray needle for real time ionization and peptide fragmentation on a Q Exactive HF Orbitrap mass spectrometer. An enhanced Fourier transform-resolution spectrum (resolution=70 000) followed by the higher-energy collisional dissociation MS/MS spectra from the 12 most intense parent ions were analyzed along the chromatographic run. Dynamic exclusion was set at 40 s.

For peptide identification, MS/MS spectra were searched with the SEQUEST-HT algorithm implemented in Proteome Discoverer. Database search was performed against the Uniprot database containing all sequences from Homo sapiens and frequently observed contaminants (HUMAN database as downloaded at 8th Nov, 2016 containing 70,877 entries), using the following parameters: trypsin digestion with 2 maximum missed cleavage sites, precursor and fragment mass tolerances of 800ppm and 0.3Da, respectively, carbamidomethyl cysteine as fixed modification and methionine oxidation as dynamic modification. Peptide identification was performed as described above.

Additionally, results files from the aforementioned Proteome Discoverer database searches were analyzed using Sequest (XCorr Only) (version IseNode in Proteome Discoverer 2.1.0.81) and X! Tandem (The GPM, thegpm.org; version X! Tandem Alanine (2017.2.1.4)) in Scaffold (Scaffold_5.0.1, Proteome Software Inc., Portland, OR). Protein identifications were accepted if there was at least one identified peptide. Proteins that contained similar peptides and could not be differentiated based on MS/MS analysis alone were grouped to satisfy the principles of parsimony. Proteins sharing significant peptide evidence were grouped into clusters.

Electrophoretic mobility shift assay (EMSA), RT-qPCR and transcriptomic assays

For EMSA analysis, HeLa cells (1.75×10^6) were seeded in 100mm dishes and cultured for 24h. At that time, the cells were exposed to 25μM IHSF115 or vehicle for 2, 4 or 6h. Additional cultures were similarly exposed for 4 or 6h, but the medium was replaced with fresh medium containing 25μM IHSF115 or vehicle at 2h or at 2h and 4h, respectively. A set of cultures was subjected to a heat treatment at 43.5^0C for 30min. Cells were harvested, and whole cell extracts were prepared as previously described (IX, X). Protein concentrations were determined by a Bradford-based protein assay. Partially complementary oligonucleotides 5´-GCCTCGAATGTTCGCGAAGTTT-3´and 5´-CGAAACTTCGCGAACATTCGAG-3´ were annealed to obtain a probe fragment containing a heat shock element (HSE) sequence (I). The fragment was labeled with [α-32P]dCTP (XI). For typical binding reactions, 5μl of cell extract containing 15μg of proteins were combined with 10μl of “Kingston” buffer (4mM MgCl2, 0.24mM EDTA, 24%(v/v) glycerol, 24mM HEPES pH7.9), 2μl of poly(dI-dC) at 1mg/ml and 1μl of water. After a 15-min pre-incubation in ice, 2μl of the labeled probe (~10 000cpm) were added, and the reaction was incubated for 15min at room temperature. Samples from cells exposed to

25µM IHSF115 for 6h (medium refreshed after 2 and 4 h) were used for HSF1 supershift. For supershift assays, binding reactions included 1µl of a rabbit anti-human HSF1 polyclonal antibody (ADI-SPA-901; Enzo Biochem) or a rat anti-mouse HSF2 monoclonal antibody (3E2) (ADI-SPA-960; Enzo Biochem). Reactions were electrophoresed on 4.5% native polyacrylamide gels. Dried gels were subjected to autoradiography.

To quantify *HSPA1A* mRNA, HeLa cells (3×10^5) were seeded in 6-well plates and cultured for 24h. At that time, cells were exposed to compound or vehicle for 2h and then harvested. Total RNA was prepared using the RNeasy Mini Kit (Qiagen, Hilden, Germany) and reverse-transcribed using Transcriptor reverse transcriptase and an anchored oligo (dT)18 primer (both from Roche). qPCR was performed using LightCycler FastStart DNA Master SYBR Green I and a LightCycler instrument (both from Roche). Quantitative expression values were extrapolated from standard curves and normalized to *β2-microglobulin (B2M)* and *glucuronidase beta* (*GUSB*) values. Specific oligonucleotides were: *HSPA1A*: 5´-ACAGGCTGGTGAACCACTTC-3´ (F), 5´-CCCTGGTGATGGACGTGTAG-3´ (R); *B2M*: 5´-CCAGCAGAGAATGGAAAGTC-3´ (F), 5´-GATGCTGCTTACATGTCTCG-3´ (R); *GUSB*: 5´-AAACGATTGCAGGGTTTCAC-3´ (F), 5´-CTCTCGTCGGTGACTGTTCA-3´ (R).

For transcriptomic analysis, HeLa cells (1.75×10^6) were seeded in 100mm dishes and cultured for 24h. At that time, cells were exposed for 6h to 25µM IHSF115 or vehicle. Medium was replaced with fresh medium containing 25µM IHSF115 or vehicle at 2h and 4h. In some experiments, 10µg/ml actinomycin D (Merck) was added 10min after compound or vehicle, and the cultures were incubated for 1h. Cells were harvested, total RNA was prepared and processed using the GeneChip WT PLUS Reagent kit (Applied Biosystems, Waltham, MA). The resulting biotinylated cRNA was hybridized with GeneChip Human Gene 2.0 ST Array (Applied Biosystems), following the manufacturer´s instructions. Arrays were scanned with a GeneChip scanner 3000 7G (Applied Biosystems). Raw data were normalized and gene levels analyzed using the RMA algorithm (Transcriptome Analysis Console Software; Applied Biosystems). Fold changes between experimental conditions were calculated as ratios of means of gene expression signals. Genes with ≥1.500-fold or ≤ 0.667-fold changes relative to the reference were included for further analysis. Data from microarray experiments were corroborated by RT-qPCR for selected genes (*DNAJA4*, *DNAJB1*, *HIST1H2AB*, *HSPA1L*, *HSPA6, EGR3*, *IER5*, *PTGS2*, *LONRF1*, *SERTM1* and *VHLL*). cDNA was prepared from total RNA using the High-Capacity RNA-to-cDNA Kit of Life Technologies (Carlsbad, CA). The cDNA was subjected to TaqMan real-time PCR using TaqMan Gene Expression Master Mix and TaqMan Gene Expression Assays (both from Life Technologies; see Supplementary Table S12 for Assay IDs) in an 7900HT Fast Real-TimePCR System (Applied Biosystems). Sequence Detector Software 2.4 (Applied Biosystems) was used for data analysis. A threshold cycle (CT) value was determined from a log-linear plot of the PCR signal versus

the cycle number. All data were converted to the linear form by 2^{-CT} determination. *B2M* (Assay ID Hs00187842 m1), *GUSB* (Assay ID Hs00939627 m1) and *HPRT1* (Assay ID Hs02800695 m1) were used as endogenous controls. All data were converted to the linear form by 2^{-CT} determination. Gene ontology analyses were based on the Database for Annotation, Visualization and Integrated Discovery (DAVID; http: //david.abcc.ncifcrf.gov) (XII).

Statistics

Unless indicated otherwise, data are presented as means, or means ± SD, of at least three independent experiments, each performed in triplicate. Data were tested for normality using Shapiro–Wilk test. All normally distributed data were analyzed using Student's t test or one-way ANOVA, followed by Dunett´s multiple comparisons test. For data not normally distributed, statistical analyses were performed using the non-parametric Kruskal–Wallis test followed by Dunn's multiple comparison test. All analyses were performed using GraphPad Prism Version 7.00. Microarray data were analyzed using the limma software included in Transcriptome Analysis Console (Applied Biosystems). The criterion for significance in statistical analyses was set at $p \leq 0.05$.

I. Vilaboa N, Boré A, Martin-Saavedra F, Bayford M, Winfield N, Firth-Clark S, et al. New inhibitor targeting human transcription factor HSF1: effects on the heat shock response and tumor cell survival. Nucleic Acids Res. 2017;45:5797-5817.

II. Xia W, Vilaboa N, Martin JL, Mestril R, Guo Y, Voellmy R. Modulation of tolerance by mutant heat shock transcription factors. Cell Stress Chaperones. 1999;4:8-18.

III. Martín-Saavedra FM, Ruíz-Hernández E, Boré A, Arcos D, Vallet-Regí M, Vilaboa N. Magnetic mesoporous silica spheres for hyperthermia therapy. Acta Biomater. 2010;6:4522-31.

IV. Cardona M, López JA, Serafín A, Rongvaux A, Inserte J, García-Dorado D, et al. Executioner Caspase-3 and 7 Deficiency Reduces Myocyte Number in the Developing Mouse Heart. PLoS One. 2015;10:e0131411.

V. Martínez-Bartolomé S, Navarro P, Martín-Maroto F, López-Ferrer D, Ramos-Fernández A, Villar M, et al. Properties of average score distributions of SEQUEST: the probability ratio method. Mol Cell Proteomics. 2008;7:1135-45.

VI. Navarro P, Vázquez J. A refined method to calculate false discovery rates for peptide identification using decoy databases. J Proteome Res. 2009;8:1792-6.

VII. Bonzon-Kulichenko E, Garcia-Marques F, Trevisan-Herraz M, Vázquez J. Revisiting peptide identification by high-accuracy mass spectrometry: problems associated with the use of narrow mass precursor windows. J Proteome Res. 2015;14:700-10.

VIII. Navarro P, Trevisan-Herraz M, Bonzon-Kulichenko E, Núñez E, Martínez-Acedo P, Pérez-Hernández D, et al. General statistical framework for quantitative proteomics by stable isotope labeling. J Proteome Res. 2014;13:1234-47.

IX. Zuo J, Baler R, Dahl G, Voellmy R. Activation of the DNA-binding ability of human heat shock transcription factor 1 may involve the transition from an intramolecular to an intermolecular triple-stranded coiled-coil structure. Mol Cell Biol. 1994;14:7557-68.

X. Zuo J, Rungger D, Voellmy R. Multiple layers of regulation of human heat shock transcription factor 1. Mol Cell Biol. 1995;15:4319-30.

XI. Vilaboa NE, Calle C, Pérez C, de Blas E, García-Bermejo L, Aller P. cAMP increasing agents prevent the stimulation of heat-shock protein 70 (HSP70) gene expression by cadmium chloride in human myeloid cell lines. J Cell Sci. 1995;108:2877-83.

XII. Huang da W, Sherman BT, Lempicki RA. Systematic and integrative analysis of large gene lists using DAVID bioinformatics resources. Nat Protoc. 2009;4:44-57.
