# Supplementary material for: Disruption of Proteostasis by Natural Products and Synthetic Compounds That Induce Pervasive Unfolding of Proteins: Therapeutic Implications
Source: Pharmaceuticals (Basel). 2023 Apr 19;16(4):616. doi: 10.3390/ph16040616 (PMC10145903; doi:10.3390/ph16040616)
Supplement: Supplementary file 1 [file pharmaceuticals-16-00616-s001.zip › Table S16 revised.pdf]

Table S16: Targeted PN components and proteins known to be ubiquitinated

PN system component	Commonly aggregating proteins	Additional proteins caused to aggregate by WA	Additional proteins caused to aggregate by IHSF115	Additional proteins caused to aggregate by IHSF058	Proteins modified by IHSF058
Heat shock response, chaperones and co-chaperones	BAG3, CRYAB, DNJA1, DNJB6, HPBP1, HSF1, HSPB1, HSPB8, SACS, TBCE	BAG5, DNJB1, DNJC2, DNJC7, HSP7E, PPID, PPIL2, TBCB, TBCD	BAG1, BAG6, DNJB1, DNJC7, HS71L, HSP72, PFD5, PPID, TBCB, TBCC	BAG1, BAG6	BAG1, 3 and 5, DJC21, DNJC2, 7 and 9, HPBP1, HS74L, HS105, HSP74, HSP7E, HSPB8, PFD1,2 and 4-6, PPIH, PPIL2, TBCB, TBCD
Proteasome	PRS6A, PSA4, PSA5, PSDE	ECM29, PCBP2, PRS6B, PRS7, PSB2, PSD10, PSD11, PSD12, PSD13, PSMD2, PSMD3, PSMD6, PSMD8, PSMG2	A0A024RA52, DCA11, PCBP2, PRS4, 6B, 7 and 8, PSA1, 7 and 7L, PSB2, 3 and 7, PSD11-13, PSMD1, 2, 3 and 5-8, PSMF1, PSMG1, WDR26	PSMF1	ADRM1, PRS10, PSD11, PSME1, PSME2, PSMF1, PSMG1, PSMG2 WDR26
Ubiquitin system	ANC2, APC1, ARMC8, BRAP, CDC20, CDC73, CR025, DESI1, DTL, FBX7, FBX30, FXL12, HECD1, HERC4, HUWE1, KLHL11, LZTR1, MAEA, MKRN2, NED4L, NS1BP, NUB1, OTUD5, PLAP, RN213, RN220, SKP2, STALP, SUMO2, TRI16, TRI32, TRI37, TRI47, TS101, UB2Q1, UB2R1, UBE2C, UBP11, UBP13, UBP47, UBP48, UBXN1, UBXN6, UBXN7, UCHL3, UEVLD, VP37A, ZFP91	APC5, APC7, ATG3, BABA1, BAP1, BRCC3, CDC16, CDC23, CDC27, CHIP, CIKS, CRBN, CTR9, FBX4, FXL18, HGS, ITCH, LTN1, NOSIP, OTUL, PIAS4, RN216, RNF14, RO52, SH3R1, TRAF2, TRI18, TRI25, TRI33, UB2D3, UB2G2, UBAC1, UBAP1, UBE3C, UBP3, UBP4, UBP15, UBR1, UCHL5, USP9X, USP9Y, VP37B, XIAP ZN598	APC5, APC7, ATG3, BABA1, BAP1, BARD1, BRCA1, BRE1A, BRE1B, CDC16, CDC23, CDC27, CHIP, CIKS, CTR9, DCAF8, DTX3L, ELOB, FBX4, HERC1, HGS, LTN1, MYCB2, NPL4, OTUL, RAD18, RO52, SH3R1, TIF1B, TRAF7, TRI25, TRI26, UB2D3, UB2E3, UB2G2, UBAC1, UBE3A, UBE3C, UBL4A, UBP5, UBP34, UBQL4, UBR1, UBR2, UBR5, UBR7, UCHL5, UFD1, VP37B, XIAP	BARD1, BRE1B, CBX4, CCAR2, DCAF8, DTX3L, ITCH, ISG15, MYCB2, NIPA, NPL4, PIAS1, RAD18, RL40, RN126, RNF14, STABP, TIF1B, TRAF2, UBP4, UBR2	ARI1, ARMC8, ATG3, BRE1A and B, CCAR2, CDC20, 27 and 73, CHIP, FXL18, ISG15, LRSM1, NIPA, NOSIP, OTUB1, OTUD5, OTUL, RBP2, RNF25, RN114, RBX1, SH3R1, SUMO1, TIF1B, TRAF2, TRI16, 18, 25 and 33, TRIPC, TS101, UB2L3, UB2V1, UBA1, UBA6, UBAP1, UBE3A, 2S, 4B and 2I, UBP2L, 5, 7, 8, 10, 15, 19, 24, 47 and 48, UBR7, UBXN1, UCHL5, USP9X, VP37A, VP37B, XIAP, ZN598

Proteins known to be ubiquitinated	AAPK1, APT, ARMC6, BRAP, BZW1, CDK1, CDK2, CND3, CSDE1, DNJA1, FACD2, HERC4, HSPB1, MAGD2, NUB1, OTUD5, PA24A, PCBP1, PCNA, PLAP, PRS6A, PSA4, PSA5, PSDE, RINI, SETB1, SND1, SPG20, TRI32, TTL12, UBXN1, UBXN7, UCHL3, VATA	AIMP2, ANM1, BOLA2, CDK4, COPA, COPE, DHB4, EHD2, ESYT1, FEN1, GEMI5, GFPT1, GUAA, IF5A1, IPO7, NRDC, PCBP2, PRS7, PSB2, PSMD2, PSMD3, RABX5, RIR1, SAR1A, SC31A, SERA, SGT1, SYEP, SYLC, TBB3, TRAD1, TRI25, TXNL1, TYSY, UBP15, UCHL5, USP9X, XPO1, XPOT, ZFY16	ANM1, ARF4, ARPC4, BAG6, BOLA2, CD123, CK054, CNDP2, COPA, COPE, COPZ1, DFNA5, GEMI5, HS71L, IF4A1, IF5A1, IPO7, IQGA1, MCM3, MCM7, ML12A, MO4L1, MRE11, MYG1, NPL4, NRDC, NSUN2, PCBP2, PRDX2, PRS4, PRS7, PRS8, PSA7, PSB2, PSMD1, PSMD2, PSMD3, PSMD7, RAD18, SAR1A, SC31A, SGT1, SIN3A, SQSTM, SYLC, TBA1A, TBB3, TBB5, TIF1B, TRAD1, TRI25, TXNL1, UBP5, UCHL5, UCK2, UFD1, XPOT, ZN281	ABCF2, BAG6, CBX4, CLH1, DYL1, FEN1, GUAA, HNRH2, HNRPF, IF4A1, MAX, MCM3, MCM7, MYG1, MRE11, NPL4, PABP1, PABP4, PMS2, PUR2, PYR1, RABX5, RAD18, RGAP1, RIR1, RNZ2, SERA, TIF1B, ZN281	AAPK1, ACLY, ACOT9, ACTN4, ADRM1, AIMP2, AMPB, AP2A1, AP2B1, ARI1, ARP3, ATX2L, BOLA2, CYBP, CBX3, CCD50, CCNT1, CND3, COPA, COPB2, CSN3, CSDE1, DDB1, DDX21, DHSO, DHX9 and 36, DNMT1, DUT, DC1I2, EIF3A, 3B, 3C, 3E and 3I, ELAV1, ESYT1, EXOS7, FA98A, FERM2, FND3B, FSCN1, G3BP1 and 2, G6PD, GEMI5, GUAA, HAT1, HDAC1 and 2, HNRPF, HPRT, HS105, HSP74, IF2B, IF6, ILF3, ILEU, IPYR2, LANC1, MAT2B, MCM6, MDHC, MK01, MTA2, MTCH2, MYOF , NCBP1, NU155, NUCL, NUFP2, NUP88, ODO1, OGT1, OTUD5, P5CS, PABP1, PAIRB, PAPS2, PA24A, PLIN3, PLST, POP1, PPM1G, PRP8, PRS10, PUR2, RENT1, RGAP1, RIR1, RL3, 4-8, 10, 13A and 36, RNZ2, RPN2, RS2 and 9, SAHH, SEC13, SF3B2, SGT1, SMC1A, SMRC1 and 2, SND1, SPART, SYDC, SYFB, SYIC, SYK, SYLC, SYRC, SYVC, TIF1B, TOM1, TRAD1, TRI25, TTL12, TWF1, TXNL1, TYSY, UBA1, UBP5 and 15, UCK2, USP9X, VATA, VPS35, WAC, XPO1, XRCC5 and 6, ZCCHV, ZFY16, ZNT1

Autophagy	ATG4B, HSPB8, PI3R4, SHLB1, SNX4**,** TPPC8, VP33A, VPS16, VPS18, VPS41	ATG3, EXOC8, PK3C3, RO52, SNX18, TAB2, TBC17, TLK2, TPPC4, VPS11, 25, 39 and 51, WDR6, WDR41, WIPI2	ARFP2, ATG3, PK3C3, RO52, SQSTM, TAB2, TBC17, TPPC4, VPS25, VPS39, VPS51, WDR41, WIPI2	DYL1, GSK3A, SNX7, TLK2	ARFP2, ATG3, 4B, 7 and 9A, HSPB8, LRSM1, PK3C3**,** TAB2, VPS39
Translation initiation	GCN1	CNOT3, EI2BB, GEMI5, IF4G2, IF4G3, IF5A1, T2EB	CNO11, CNOT3, CNOT8, EIF3F, IF2M, IF4A1, IF4A2, IF5A1	EI2BB, EIF3F, IF4A1, IF4A2, IF4G2, T2EB	4EBP1, CNO11, CNOT3, EI2BA, EI2BB, EIF3A, 3B, 3C, 3D, 3E, 3H, 3I, 3K, 3L and 6, GEMI5, IF2P, 4B, 4E, 4E2, 4G1 and 4G3
